# A live-attenuated pneumococcal vaccine elicits CD4^+^ T-cell dependent class switching and provides serotype independent protection against acute otitis media

**DOI:** 10.1002/emmm.201202150

**Published:** 2013-11-04

**Authors:** Jason W Rosch, Amy R Iverson, Jessica Humann, Beth Mann, Geli Gao, Peter Vogel, Michael Mina, Kyle A Murrah, Antonia C Perez, W Edward Swords, Elaine I Tuomanen, Jonathan A McCullers

**Affiliations:** 1 Department of Infectious Diseases, St. Jude Children's Research HospitalMemphis, TN, USA; 2 Department of Pathology, St. Jude Children's Research HospitalMemphis, TN, USA; 3 Emory University School of MedicineAtlanta, GA, USA; 4 Department of Microbiology and Immunology, Wake Forest School of MedicineWinston-Salem, NC, USA

**Keywords:** otitis media, *Streptococcus*, vaccine, virulence

## Abstract

Acute otitis media (AOM) caused by *Streptococcus pneumoniae* remains one of the most common infectious diseases worldwide despite widespread vaccination. A major limitation of the currently licensed pneumococcal vaccines is the lack of efficacy against mucosal disease manifestations such as AOM, acute bacterial sinusitis and pneumonia. We sought to generate a novel class of live vaccines that (1) retain all major antigenic virulence proteins yet are fully attenuated and (2) protect against otitis media. A live vaccine candidate based on deletion of the signal recognition pathway component *ftsY* induced potent, serotype-independent protection against otitis media, sinusitis, pneumonia and invasive pneumococcal disease. Protection was maintained in animals coinfected with influenza virus, but was lost if mice were depleted of CD4^+^ T cells at the time of vaccination. The live vaccine induced a strong serum IgG2a and IgG2b response that correlated with CD4^+^ T-cell mediated class switching. Deletion of genes required for microbial adaptation to the host environment is a novel live attenuated vaccine strategy yielding the first experimental vaccine effective against pneumococcal otitis media.

## Introduction

Acute otitis media (AOM) and sinusitis are two of the most common infectious diseases of children and the most common infections for which antibiotics are prescribed in the United States (Coco *et al*, 2010; Rodgers *et al*, 2009). *Streptococcus pneumoniae* is the leading bacterial cause of AOM (Rodgers *et al*, 2009). Purified polysaccharide vaccines (PPV) directed against *S. pneumoniae* do not elicit antibodies in children under age 2 and exhibit limited protection against AOM and sinusitis in any age group (Lottenbach *et al*, 1999). Pneumococcal conjugate vaccines are highly protective against invasive disease but are not effective against AOM or sinusitis (Eskola *et al*, 2001). A reduction in carriage of vaccine serotypes of *S. pneumoniae* following widespread use of the 7-valent pneumococcal conjugate vaccine [Prevnar 7 (PCV7)] has been linked to decreased office visits and antibiotic prescriptions for AOM through herd immunity (Grijalva *et al*, 2006; Shea *et al*, 2011), although similar trends have not been seen for acute bacterial sinusitis (Shapiro *et al*, 2011). Despite some success in reducing the clinical impact of *S. pneumoniae* in children through vaccination, the burden of disease related to pneumococcal AOM and sinusitis remains significant (Coker *et al*, 2010; Taylor *et al*, 2012).

Traditional vaccines targeting the polysaccharide capsule of *S. pneumoniae* are thought to work through generation of antibodies that bind capsule and facilitate opsonophagocytosis. Since purified polysaccharide does not elicit T-cell responses, CD4^+^ T-cell help for isotype class switching and development of memory B cells is absent. Conjugation of polysaccharide to protein carriers overcomes this defect, improving memory responses and increasing immunogenicity in children under 2 years of age (Knuf *et al*, 2011). Serum immunoglobulin G (IgG) of the IgG2 isotype is the predominant antibody produced by vaccination with both PPVs and PCVs, although there is a shift towards IgG1 in responses of young children to PCV (Lottenbach *et al*, 1999). Neither vaccine produces significant amounts of IgA, the predominant antibody at the mucosal surface that is the site of pneumococcal colonization and otitis media. We sought to determine if a live, attenuated vaccine against *S. pneumoniae* could overcome these limitations and more specifically, protect against otitis media.

Concepts for vaccines active at mucosal sites have focused on nasopharyngeal colonization as a critical endpoint. It is generally assumed that decreased colonization, the first step in pneumococcal pathogenesis, translates to decreased development of all diseases. However, mucosal vaccines shorten the duration of colonization but do not prevent it entirely. Therefore, the relative kinetics of development of disease at different sites *versus* development of a protective response in that site would impact vaccine efficacy. Evidence is strong that interruption of colonization protects against invasive disease. For example, intranasal application of live, attenuated *S. pneumoniae* mediates a potent, serotype-independent mucosal and systemic immune response that attenuates subsequent carriage in the nasopharynx and protects against invasive challenge (Roche *et al*, 2007) (Cohen *et al*, 2012). Intranasal application of an unencapsuled killed whole cell vaccine with cholera toxin adjuvant also protected against colonization (Malley *et al*, 2005). Administration of avirulent pneumococci by intraperitoneal injection was found to be non-protective against hetereologous challenge (Chimalapati *et al*, 2011) whereas administration of attenuated pneumococci via intranasal inoculation conferred effective protection (Kim *et al*, 2012). In no case, however, has it been tested if these vaccines that shorten but do not completely prevent colonization also impact AOM or pneumonia.

Previously studied live attenuated strains of *S. pneumoniae* may not be optimal vaccine candidates because they were generated by deleting several important, highly immunogenic virulence factors. These virulence genes include important antigens that induce potent antibody responses following pneumococcal carriage and otitis media in young children (Melin *et al*, 2008; Rapola *et al*, 2000; Simell *et al*, 2001). As an alternative approach, we focused on candidate genes essential for microbial adaptation to the host environment while maintaining a full set of virulence determinants. Deletion of *ftsY*, a central component of the signal recognition particle (SRP) pathway that is responsible for delivering membrane and secretory proteins to the proper cellular destination, is lethal to many bacterial species but is tolerated in streptococci (Crowley *et al*, 2004; Rosch *et al*, 2008b). Mutants in the SRP pathways show heightened sensitivity to environmental stresses and have greatly diminished virulence, though many traditional virulence factors are still produced (Rosch *et al*, 2008b; Trevino *et al*, 2010). Deletion of *caxP*, a calcium/magnesium transporter that is essential for both bacterial colonization and invasive disease, renders host physiological conditions in the blood and mucosa toxic to the bacterium due to impaired cation transport properties (Neef *et al*, 2011; Rosch *et al*, 2008a). Strains lacking one of these genes may make ideal live vaccine candidates as they either colonize but do not cause invasive disease (*ftsY*-), or are rapidly cleared from the mucosa and do not cause invasive disease (*caxP*-). Hence, these mutants represent a novel approach of targeting non-traditional virulence determinants to generate a highly attenuated bacterial strain. In this study, we tested these live attenuated candidates as vaccines against a variety of mucosal and invasive pneumococcal diseases in mouse models. The *ftsY*- vaccine candidate in a serotype 19F background was highly immunogenic and protected mice against AOM, sinusitis, invasive disease, and secondary bacterial pneumonia following influenza in a serotype independent manner.

## Results

### Attenuation by preventing bacterial adaptation to the host environment

**Figure 1:**
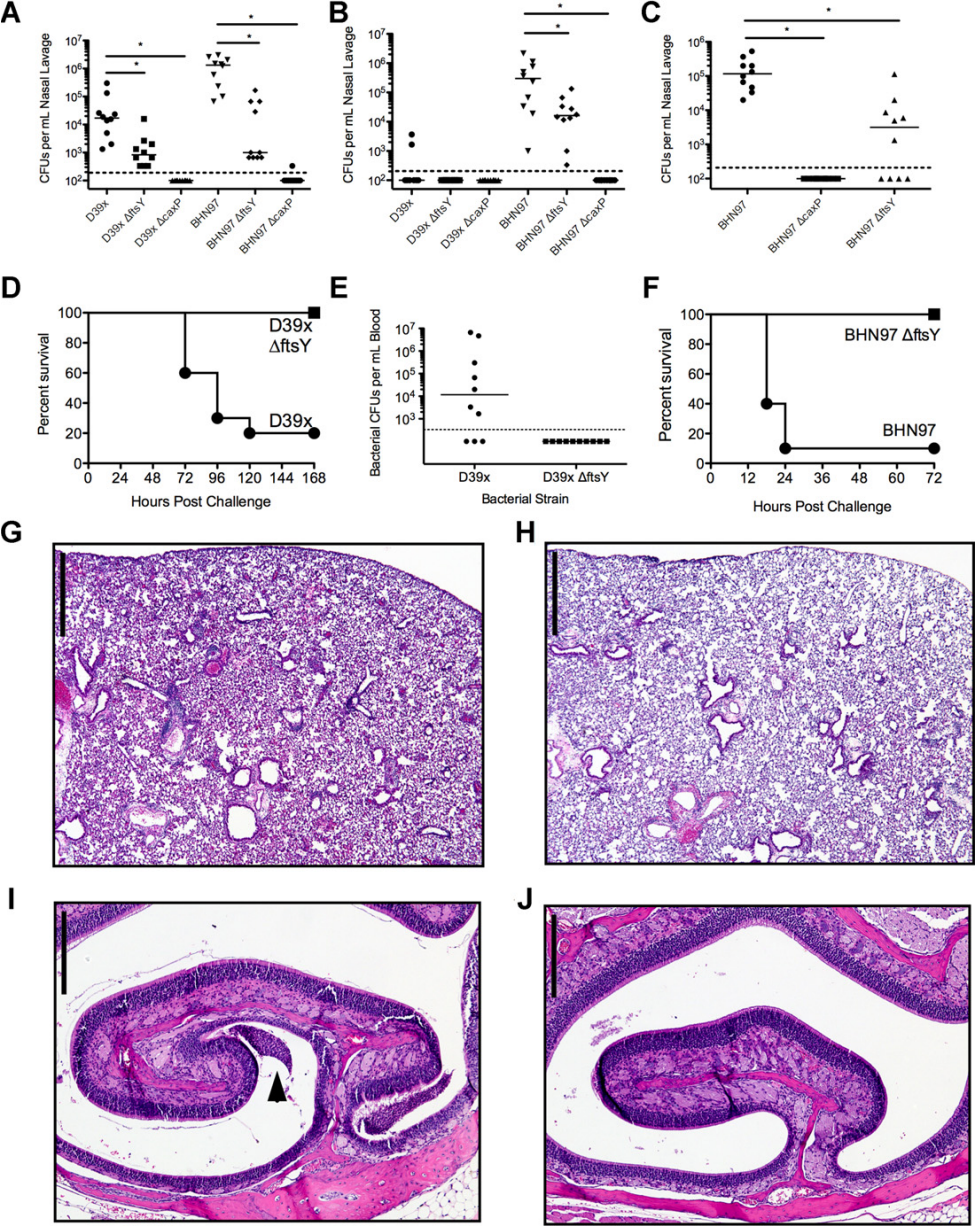
Characterization of attenuated live vaccine strains. A–C  Duration of nasal colonization by wild-type and vaccine strains was measured in nasal lavages plated for CFUs on days 1, 3 and 7 postinoculation. *N* = 10 mice per group; values are mean ± SEM, * = *p* < 0.05 by Mann–Whitney. D, E  Comparison of mice challenged intranasally with parental D39x strain or D39Δ*ftsY* for (D) survival (*N* = 10 mice per group) and (E) presence of bacteria in blood 48 h postinfection. Each symbol is a mouse; *n* = 10 per group. F  Comparison of mice infected intraperitoneally with BHN97 or BNH97Δ*ftsY* and monitored for survival. G–J  Comparison of lung (G, H) and ear (I, J) histopathology in mice challenged intranasally with parental BHN97 (G, I) or BNH97Δ*ftsY* (H, J). In the nasal passages, mucopurulent exudate is present only in the BHN97 infected mice (arrows in I). Scale bars for lungs are 600 μm and for nasal sections 250 μm.

The relevant characteristics of the vaccines tested in this study are detailed in Table 1. For the live vaccine candidates, we utilized two background strains, the invasive serotype 2 strain D39x that causes pneumonia and sepsis, and the noninvasive serotype 19F strain BHN97 which normally causes sinusitis/purulent rhinitis and AOM. In each of these backgrounds, we generated two separate mutants using deletions targeting *ftsY* and *caxP* and tested their virulence in terms of colonization of the nasopharynx and invasive disease. Deletion of *caxP* in both strain backgrounds resulted in elimination of an intranasal inoculum of 10^5^ bacteria from the nasopharynx within 24 h (Fig 1A). The *ftsY* deletion mutants were able to colonize for at least 24 h but with significantly reduced titers compared to the parental strain (Fig 1A). The BHN97ΔftsY strain had the longest colonization duration of any of the mutants, with measurable titers out to seven days as opposed to the other strains (Fig 1B,C). Deletion of *caxP* has previously been shown to completely attenuate pneumococcus for invasive disease (Rosch *et al*, 2008a). Deletion of *ftsY* in D39x background prevented translocation into the bloodstream and mortality compared to the parental D39x (Fig 1D,E). Deletion of*ftsY* in the BHN97 strain rendered the bacteria unable to cause infection when administrated by intraperitoneal injection (Fig 1F). Administration of the BHN97 *ftsY* deletion via the intranasal route resulted in marked decreases in both lung and sinus inflammation compared to the parental strain (Fig 1G–J). The deletion of either *caxP* or *ftsY* resulted in no loss of the expression of the antigenic virulence proteins pneumolysin, CbpA, or PspA (supplementary Fig S1). Interestingly, we observed a consistent trend whereby the *ftsY* mutant expressed greater amounts of both CbpA and PspA compared to the parental wild type strain. These data support the contention that these strains are sufficiently defective in both mucosal and invasive disease to warrant further consideration as live vaccine candidates.

**Table 1:** Vaccines used in this study

Vaccine	Serotype(s)	Relevant characteristics
D39ΔftsY	2	Live, attenuated, colonizing strain, heterologous challenge^a^
D39ΔcaxP	2	Live, attenuated, non-colonizing strain, heterologous challenge
BHN97ΔftsY	19F	Live, attenuated, colonizing strain, homologous challenge
BHN97ΔcaxP	19F	Live, attenuated, non-colonizing strain, homologous challenge
PCV7	4, 6B, 9V, 14, 18C, 19F, and 23F	Multivalent, conjugated polysaccharide vaccine, homologous challenge
PCV13	1, 3, 4, 5, 6A, 6B, 7F, 9V, 14, 18C, 19A, 19F, and 23F	Multivalent, conjugated polysaccharide vaccine, homologous challenge
PPV23	1, 2, 3, 4, 5, 6B, 7F, 8, 9N, 9V, 10A, 11A, 12F, 14, 15B, 17F, 18C, 19A, 19F, 20, 22F, 23F and 33F	Multivalent polysaccharide vaccine, homologous challenge
Mock	None	Carrier only as baseline control

a Vaccination is homologous or heterologous to the serotype 19F, AOM- and sinusitis-causing challenge strain.

### A live, attenuated vaccine protects against otitis media and sinusitis

**Figure 2:**
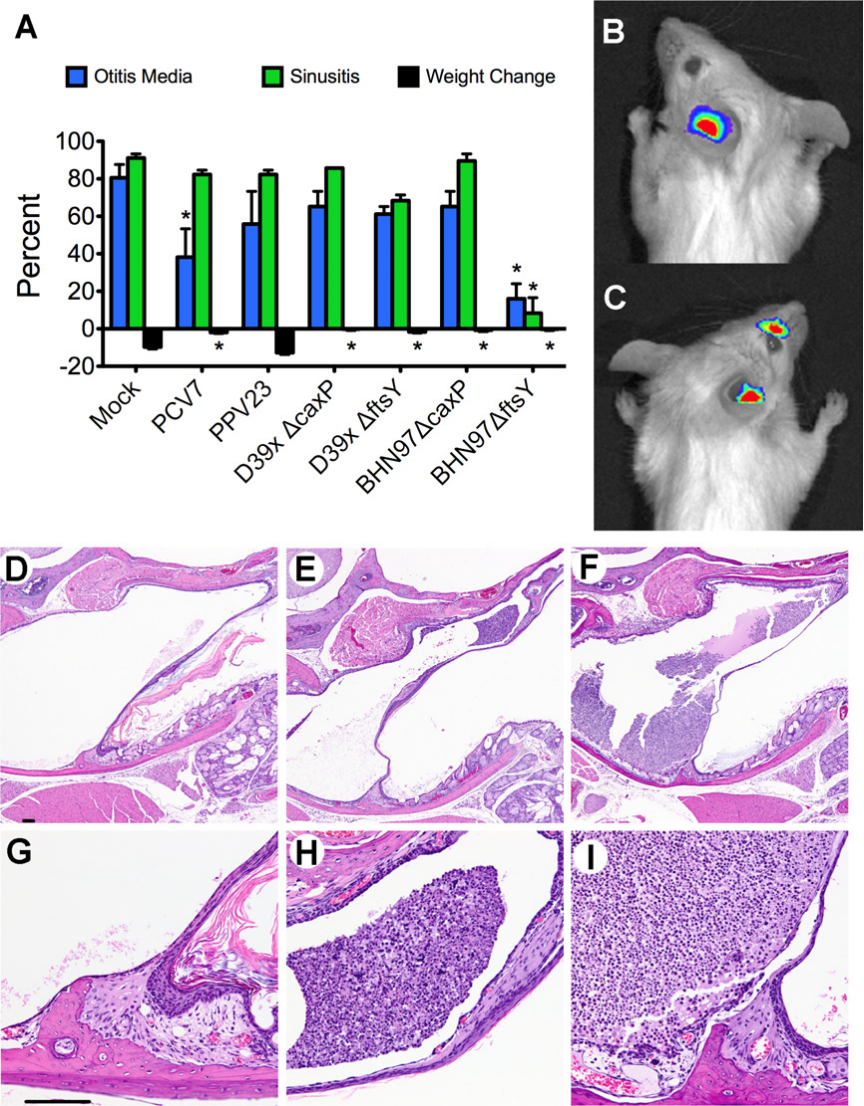
Vaccine protection against otitis media and sinusitis. Mice (*n* = 25–31 per group, performed at least twice for each group) were mock-vaccinated with PBS (Mock) or vaccinated with live attenuated vaccines deleted for *caxP* or *ftsY* on either a type 2 (D39Δ*caxP*, D39Δ*ftsY*) or type 19F (BNH97Δ*caxP*, BNH97Δ*ftsY*) background. Mice were challenged with a bioluminescent *S. pneumoniae* strain BNH97X (type 19F) and imaged twice daily for development of AOM or sinusitis. A  The proportion of mice developing an infection of the ear or sinus by Xenogen imaging. * = *p* < 0.05 by Chi-squared test compared to the mock vaccinated group. PPV23 was used as a negative control (60% otitis and 80% sinusitis). Errors bars represent standard error of the mean. B, C  Representative pictures from bioluminescent imaging of mice with (B) AOM and (C) both AOM and sinusitis are shown. D–I  Representative histopathology at 4× (top row) and 40× (bottom row) of (D, G) a normal ear, (E, H) a mouse with mild AOM and (F, I) a mouse with marked AOM are shown. Data representative of at least 25 mice per group, performed in duplicate.

To test vaccine efficacy against otitis media and sinusitis, mice were vaccinated intranasally, boosted twice and then challenged with bioluminescent BHN97 intranasally (McCullers *et al*, 2007; Smith *et al*, 2007) and followed twice daily by Xenogen bioluminescent imaging. BHN97 rapidly caused AOM in approximately 80% of naive animals within 24 h of inoculation. Sinusitis developed at the same time or shortly after the otitis infection in most mice, typically peaking within 72 h after challenge. Subsets of mice predicted by Xenogen bioluminescent imaging to be either AOM or sinusitis positive were euthanized to confirm disease by histopathology. The incidence of otitis media was significantly (*p* < 0.05 compared to mock) lower in BHN97Δ*ftsY*-vaccinated mice (Fig 2A). The other live, attenuated vaccines did not confer any significant degree of protection. Intranasal administration of heat-killed pneumococci also did not confer significant protection compared to mock animals (data not shown). Only the BHN97Δ*ftsY* vaccine significantly decreased the incidence of sinusitis (*p* < 0.05). Measurement of the total luminescence of the ears and sinuses at 24 and 72 h, respectively, confirmed the protection engendered by the BHN97Δ*ftsY* vaccine (supplementary Fig S2). Representative bioluminescent images (Fig 2B, C) and histopathology (Fig 2D–I) are pictured. Consideration of*caxP*-based mutants in protective studies was halted at this point and further experimentation went forward with the BHN97Δ*ftsY* vaccine candidate alone.

**Figure 3:**
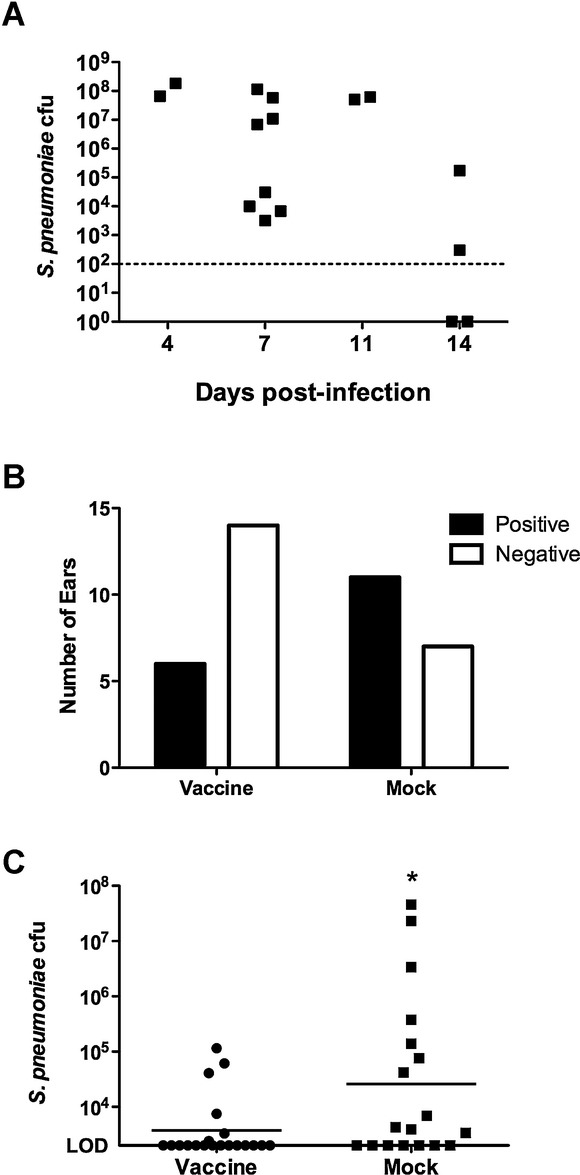
Vaccine protection in a chinchilla model of otitis media. A  The BHN97 strain is capable of causing otitis media in chinchillas via intranasal administration as observed by recoverable bacterial colony forming units (CFUs) from the middle ear (A) following challenge. B, C  Following vaccination, a reduction in the number of culture positive ears in the vaccinated group compared to the mock animals was observed (B) as well as a significant reduction in recoverable CFUs from the middle ear at 7 days postchallenge (C). * = *p* < 0.05 by Mann–Whitney.

We next sought to determine if the BHN97Δ*ftsY* vaccine confers protection in an additional animal model of experimental AOM. We opted for the chinchilla model due to the extensive characterization of this system for investigating bacterial otitis media. We established that the BHN97 strain was able to cause AOM in chinchillas when administered by the intranasal route by both otoscopy scores (data not shown) and by recovery of bacteria from the middle ear (Fig 3A). Chinchillas were vaccinated intranasally with the BHN97Δ*ftsY* vaccine and subsequently challenged with the BHN97 strain. The BHN97Δ*ftsY* vaccinated animals had a decreased incidence of culture positive ears and displayed a significant decrease in the amount of recoverable bacteria from the middle ear (Fig 3C, D). These data indicate that the BHN97Δ*ftsY* vaccine confers effective protection against AOM both in murine and chinchilla model systems.

### A live, attenuated vaccine protects against heterologous challenges

**Figure 4:**
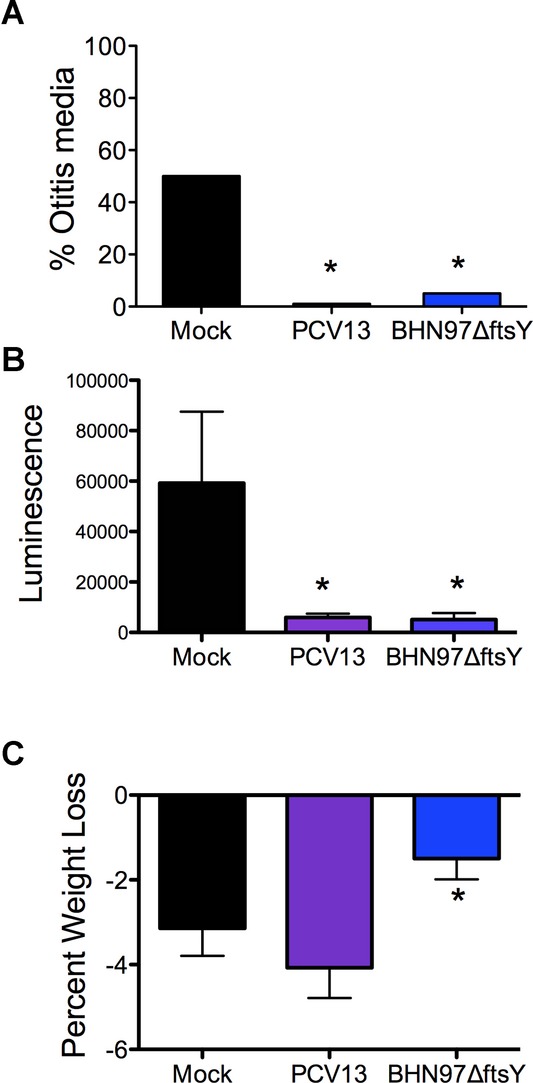
Vaccine protection against heterologous challenge. Mice (*n* = 20 per group, performed in duplicate) were mock-vaccinated with PBS (Mock) or vaccinated with PCV13 or a live attenuated vaccine deleted for FtsY on a type 19F background (BNH97Δ*ftsY*). A  Mice were challenged with a bioluminescent version of *S. pneumoniae*strain BNH54 (type 7F) and assessed by imaging for development of otitis media over 72 h (24 h time point is pictured). B, C  Quantification of the luminescent signal (B) and weight loss (C) observed in the animals further supported the observed protection. An asterisk (*) indicates a significant difference (*p* < 0.05) by Chi-squared test compared to the mock vaccinated group. PCV13 contains type 7F antigen, so this was a homologous challenge for the PCV13 group but a heterologous challenge for the BNH97Δ*ftsY* group.

We next sought to determine whether heterologous protection was conferred by the BHN97Δ*ftsY* vaccine using a serotype 7F strain of pneumococcus (BHN54) that causes AOM in approximately 50% of infected animals. The BHN97Δ*ftsY*-vaccinated animals had a ten fold lower incidence of AOM (50% AOM in mock *versus* 5% AOM in BHN97Δ*ftsY*-vaccinated *p* < 0.05) than did the mock-vaccinated animals (Fig 4A). This was confirmed by bioluminescence: Mock animals had an average RLU of 60,000 *versus* BHN97Δ*ftsY* having 5000 (Fig 4B), as well as significantly reduced weight loss (Fig 4C).

**Figure 5:**
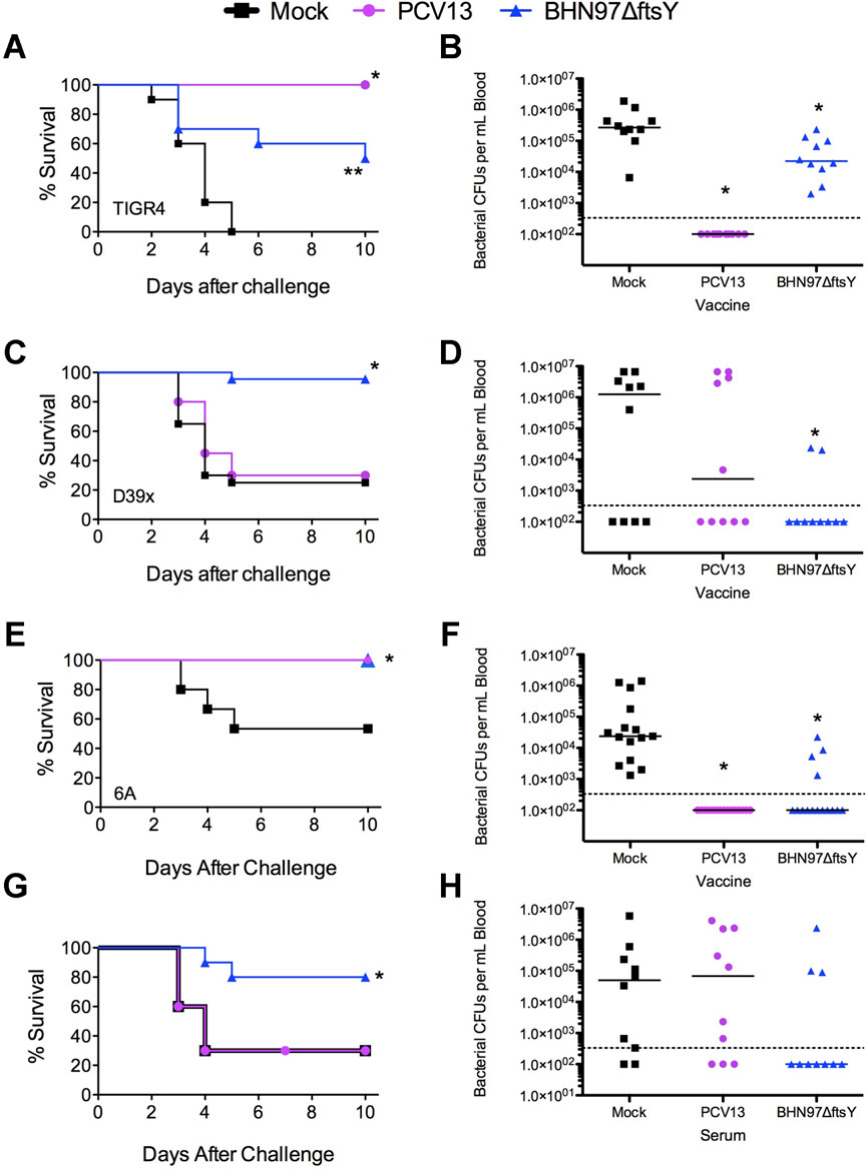
Vaccine protection from invasive infection. Mice (*n* = 10 per group) were vaccinated with PBS (Mock, negative control, black squares), PCV13 (positive control, purple circles) or a live attenuated BNH97Δ*ftsY* (type 19F, blue triangles). A–F  Mice were challenged with bioluminescent *S. pneumoniae* strains (A, B) TIGR4 (type 4) or (C, D) D39X (type 2), or (E, F) 6A4 (type 6A) and followed for survival. * = *p* < 0.05 by log rank test on the Kaplan–Meier data compared to the other 2 groups; ** = a significant difference compared to the mock vaccinated group. G, H  Passive protection (survival and blood CFU) administering mock, PCV13 or BNH97Δ*ftsY* mouse antiserum immediately prior to challenge with D39 showed no protection by PCV13 antiserum against heterologous challenge and significant protection (*) by BNH97Δ*ftsY*against heterologous challenge.

To determine the protection by the live vaccine in invasive disease, we next sought to determine the efficacy of BHN97Δ*ftsY* against lethal, heterologous pneumococcal challenges. Vaccinated mice were challenged with D39x (type 2), TIGR4 (type 4) or 6A4 (serotype 6A) and followed for mortality. Although all three challenge strains are heterologous to the BHN97Δ*ftsY* vaccine, vaccination with BHN97Δ*ftsY* resulted in significant protection against sepsis and death for all challenges compared to mock vaccination (Fig 5A, C, E). The BHN97Δ*ftsY* vaccine resulted in significantly decreased bacterial titers in the blood following infection for all three challenge strains (Fig 5B, D, F). In addition to conferring protection against AOM and sinusitis, the BHN97Δ*ftsY*-vaccinated animals showed significant decreases in bacterial nasal colonization following challenge with either heterologous or homologous challenge strains (supplementary Fig S3). We conclude that the BHN97Δ*ftsY* vaccine confers effective serotype-independent protection against AOM and invasive disease.

### A live, attenuated vaccine protects against secondary bacterial pneumonia

**Figure 6:**
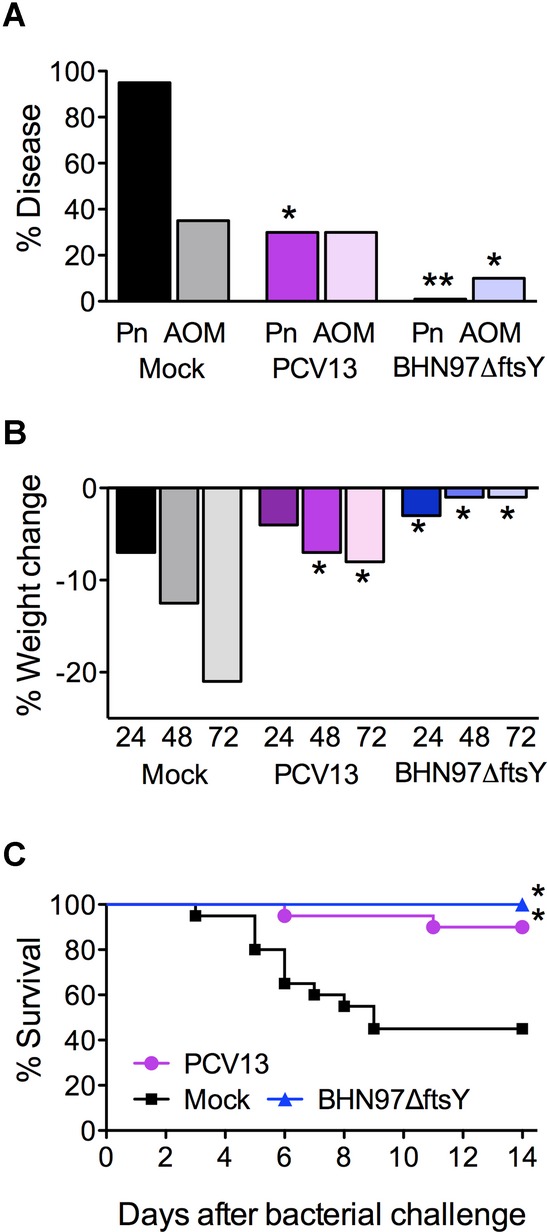
Vaccine protection for secondary bacterial co-infections after influenza. Mice (*n* = 20 per group) were mock-vaccinated with PBS (Mock), PCV13 or a live attenuated vaccine BNH97Δ*ftsY*. Mice were infected with sublethal dose of influenza virus, then challenged 7 days later with a bioluminescent *S. pneumoniae* strain BHN97X (type 19F); this is a homologous challenge for both vaccine groups. A–C  Mice were followed for (A) development of AOM or pneumonia (Pn) at 24 h as measured by bioluminescent imaging, (B) weight loss and (C) survival. * = significant difference *p* < 0.05 by (A) Chi-squared test (B) ANOVA and (C) log-rank test compared to the mock vaccinated group; ** = significant difference by Chi-squared test compared to the both other groups.

It is increasingly recognized that prior influenza infection is a major predisposing factor to bacterial pneumonia and invasive pneumococcal disease (McCullers, 2006). We modelled this synergism by administering a sublethal dose of influenza virus that caused a mild transient pulmonary infection without systemic disease followed by bacterial challenge that is not lethal without prior viral infection (McCullers & Rehg,^2002^). At post-viral infection day 7, mice were challenged with BHN97 and monitored for the development of disease by bioluminescent imaging. Within 24 h of pneumococcal challenge, 90% of the mock-vaccinated animals had developed pneumonia. The BHN97Δ*ftsY* vaccine completely prevented the development of pneumonia (Fig 6A). In this experimental synergy model, the incidence of AOM in the mock-vaccinated mice was ∼40%; however, the BHN97Δ*ftsY* vaccine reduced otitis incidence to 15% (*p* < 0.05). BHN97Δ*ftsY* was also effective in preventing weight loss as a measure of systemic illness at 24, 48 and 72 h after infection (Fig 6B). The BHN97Δ*ftsY* vaccine prevented mortality in this synergistic model of secondary bacterial infection (Fig 6C). We conclude that the effectiveness of the BHN97Δ*ftsY* vaccine is retained in the setting of prior viral infection.

### A live, attenuated vaccine elicits a distinct antibody response that is dependent on CD4^+^ T cells

**Figure 7:**
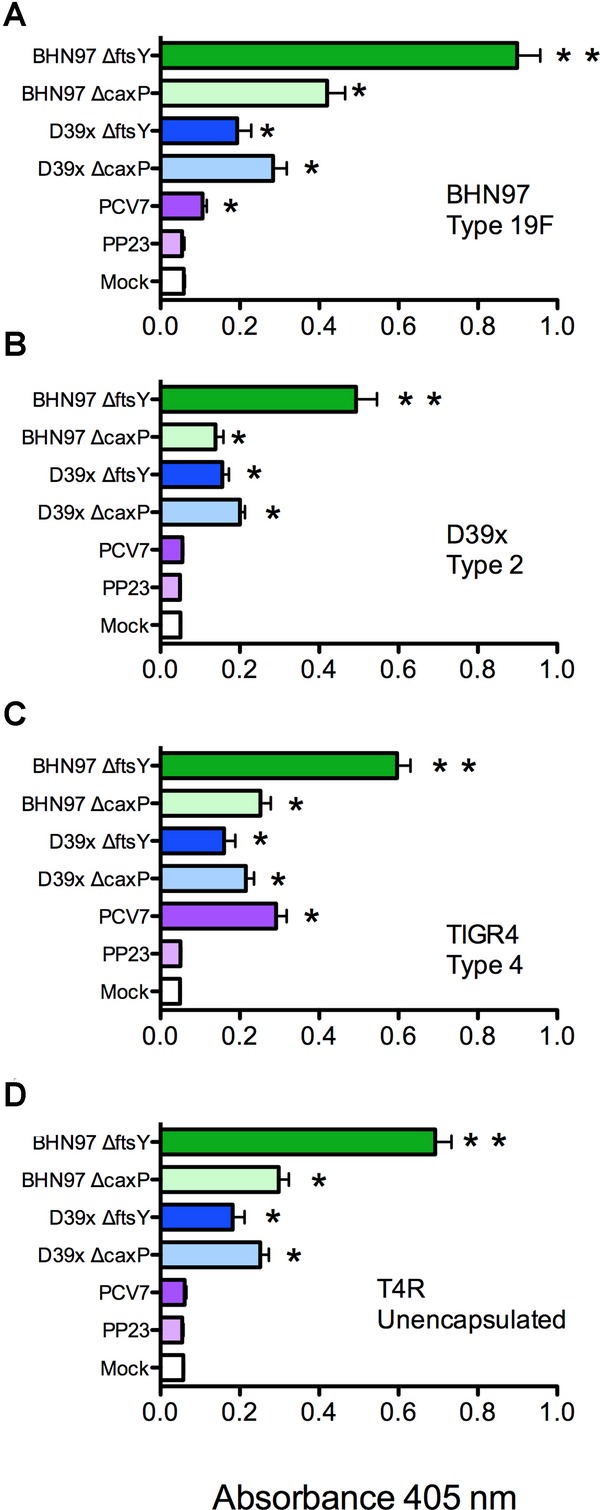
Antibody responses following vaccination. Mice (*n* = 25–31 mice per group) were vaccinated with PBS (Mock), PCV7, PPV23 or live attenuated vaccines deleted for *caxP* or *ftsY* on either a type 2 (D39Δ*caxP*, D39Δ*ftsY*) or type 19F (BNH97Δ*caxP*, BNH97Δ*ftsY*) background. A–D  Serum was tested by ELISA using whole pneumococci as antigens from strains (A) BHN97, (B) D39x, (C) TIGR4 or (D) T4R (unencapsulated version of TIGR4). The antibody dilution utilized was 1:450 for all strains. Mean OD_450_ values ± SEM are displayed. * = Significant difference *p* < 0.05 by ANOVA compared to the mock and PPV23 groups; ** = significant difference compared to all other groups. PCV7 contains type 4 and 19F antigens but not type 2; PPV23 contains all three antigens. Data representative of at least 25 mice per group, performed in duplicate.

Despite very different protective activities in disease models, the live vaccines from both the BHN97 and D39x parent strains generated high levels of antibodies reactive against pneumococcus in a serotype-independent manner (Fig 7, p < 0.05 compared to mock). The BHN97Δ*ftsY*vaccine elicited significantly higher titers of anti-pneumococcal antibodies compared to all other vaccines, independent of the capsular serotype of the test strain (Fig 7). The BHN97Δ*ftsY* vaccine also elicited antibody responses against pneumolysin, CbpA and PspA at significantly higher levels than intranasally administered heat-killed BHN97 (supplementary Fig S4). In addition, even though the BHN97 strain colonizes at high levels up to 4 weeks following administration (data not shown), the repeated inoculation of the BHN97Δ*ftsY* vaccine resulted in greater heterologous antibody titers than prolonged colonization with the parental strain (supplementary Fig S5). We conclude that the live, attenuated vaccine BHN97Δ*ftsY* is significantly more immunogenic than comparator vaccines in mice, and that the immune responses to this candidate are not serotype specific. We next undertook passive protection studies to support whether the antibody response correlated with protection. Mice were given a single injection of either mock or BHN97Δ*ftsY* serum 1 h prior to infection with D39x (Fig 3G, H). The BHN97Δ*ftsY* serum was able to confer protection against subsequent D39x challenge. We conclude that BHN97Δ*ftsY* protection is at least in part antibody-mediated.

**Figure 8:**
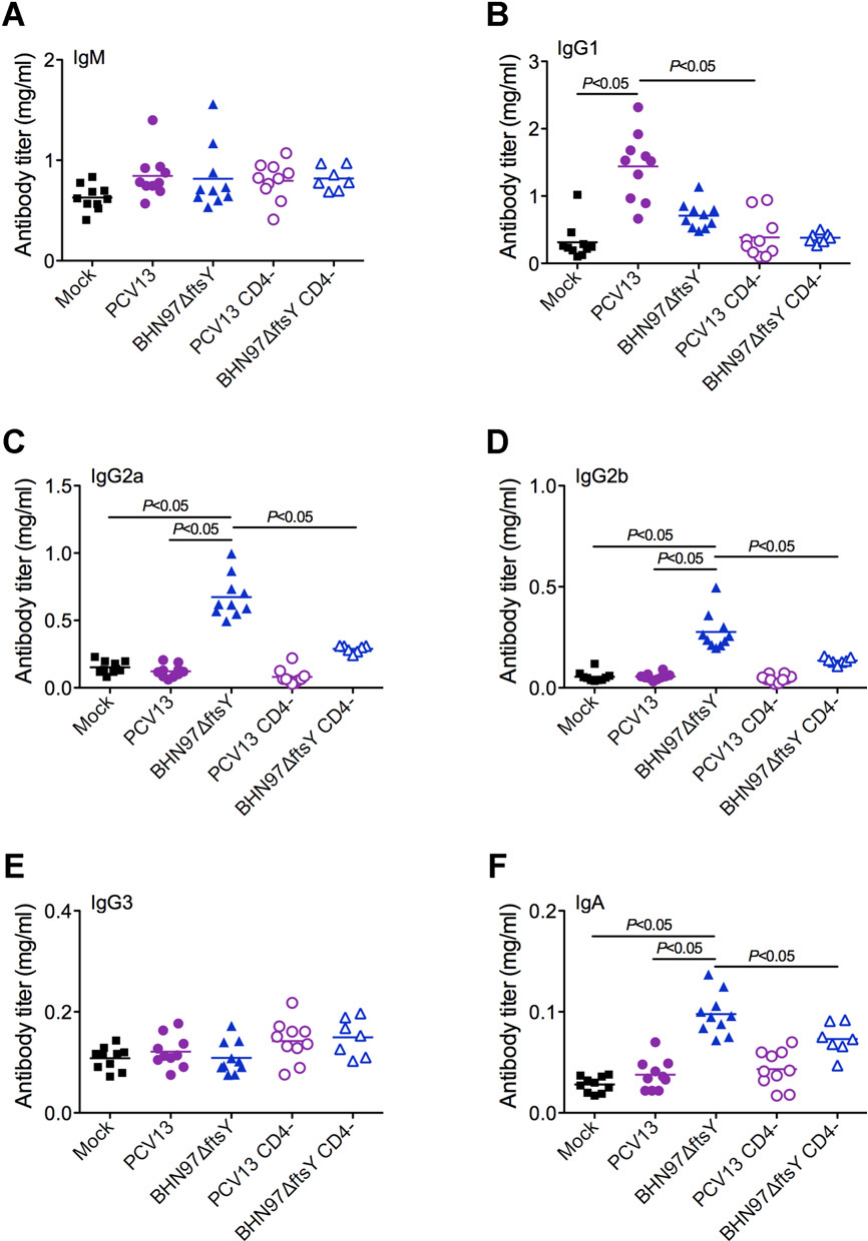
Vaccination with a live attenuated strain of *S. pneumoniaeelicits* distinct antibody isotypes. Mice (*n* = 10) were vaccinated with PBS (Mock), PCV13 or a live attenuated vaccine BNH97Δ*ftsY*. Two additional groups of mice were depleted of CD4^+^ T cells prior to vaccination. A–F  Serum antibody levels to isotypes (A) IgM, (B) IgG1, (C) IgG2a, (D) IgG2b, (E) IgG3 and (F) IgA were determined by ELISA. Antibody titers were compared between groups by ANOVA; significant differences between groups (*p* < 0.05) are highlighted in the figure. Horizontal bars within the clusters of symbols represent the mean of the group titers. Note that the scale bars are different for each antibody isotype.

To address the potential role of antibody isotypes in protection and begin to understand the mechanism of enhanced protection from the BHN97Δ*ftsY* vaccine, we measured the levels of total immunoglobulin isotypes in mouse serum 96 h following challenge. Since it is known that conjugate vaccine responses are dependent on CD4^+^ T-cell help, in some experiments we depleted CD4^+^ T cells from groups of mice at the time of vaccination to abrogate these cells' contribution to development of immunity. The antibody response to Prevnar 13 (PCV13) was dominated by IgG1; significant levels of IgM, IgA or other IgG isotypes were not seen (Fig 8). As expected, this response was abrogated in the absence of CD4^+^ T cells (Fig 8B). In contrast, the BHN97Δ*ftsY* vaccine did not significantly induce IgG1 production in vaccinated animals. Immune responses to BHN97Δ*ftsY* were shifted towards the production of IgG2a, IgG2b and IgA (Fig 8). Each of these responses required the presence of CD4^+^ T cells during vaccination. No significant differences were observed in any of the animals in terms of IgM or IgG3 levels.

**Figure 9:**
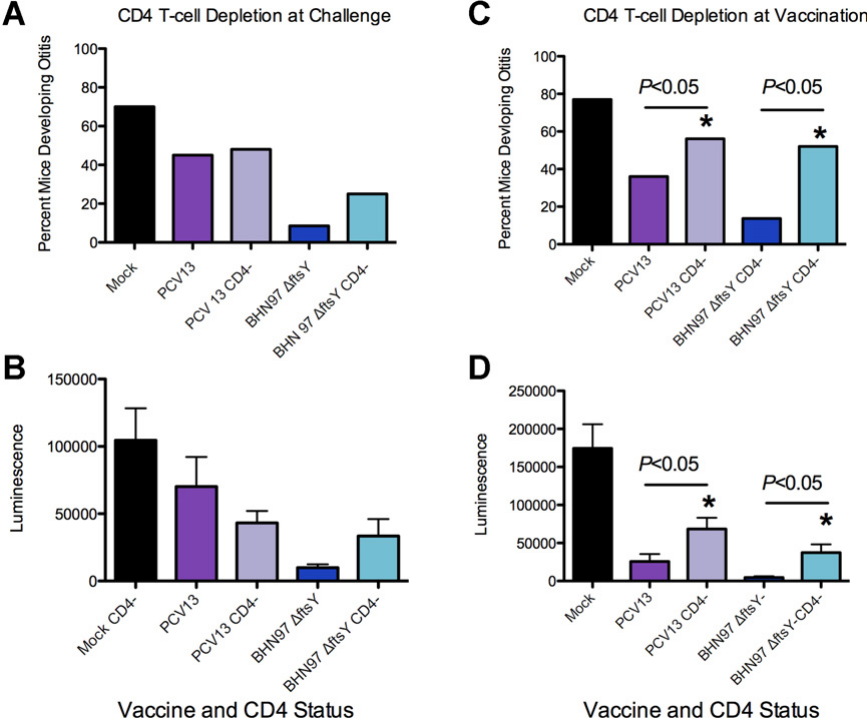
Induction of protection against acute otitis media (AOM) is CD4^+^T-cell dependent. Mice (*n* = 10) were vaccinated with PBS (Mock), PCV13 or a live attenuated vaccine BNH97Δ*ftsY*. A–D  Two additional groups of mice were depleted of CD4^+^ T cells prior to challenge (A and B) or vaccination (C and D). Mice were challenged with a bioluminescent *S. pneumoniae* strain BNH97X (type 19F) and assessed by imaging for development of AOM at 24 h. * = Significant difference *p* < 0.05 by Chi-squared test of the proportion of the non-depleted group with AOM compared to the matching group that was depleted of CD4^+^ T cells (CD4^−^).

This isotype switch to the IgG2 and IgA pattern correlated with the degree of protection against AOM, suggesting that the response elicited to the live vaccine is the more optimal antibody isotype distribution to engender protection against AOM, although we cannot eliminate the potential for additional cellular factors to be involved in protective capacity. We therefore investigated the relative contribution of CD4^+^ T cells in the development of the mucosal protection that is conferred by the BHN97Δ*ftsY* vaccine. Mice that had been depleted of CD4^+^ T cells during vaccination had higher incidences of AOM than did mice with intact CD4^+^ T cells during vaccination, indicating that CD4^+^ T-cell help is required for an effective response from these vaccines (Fig 9). Depletion of CD4^+^ T cells at the time of challenge did not have a statistically significant effect on protection, though modest increases in the incidence and severity of AOM were observed suggesting these cells may also play a direct role in protection (Fig 9). We conclude that CD4^+^ T-cell-dependent isotype switching is one factor required for protection from AOM when mediated by the live, attenuated pneumococcal vaccine BHN97Δ*ftsY*.

## Discussion

Experimental trials of vaccines against *S. pneumoniae* began in the early 1910s to combat epidemic pneumococcal pneumonia, which had a 35% case fatality rate in gold miners in South Africa (Kazanjian, 2004). The first PPV was licensed in 1977, and the first PCV in 2000. The PPV was updated in 1983 to cover 23 serotypes, while the conjugate vaccine was updated to 13 serotypes in 2010 (Nunes & Madhi, 2011). PPV is modestly effective at preventing invasive disease in adults, although most studies were done in populations with high carriage rates (Mangtani *et al*, 2003). The development of PCVs was a major advance in protection against invasive disease, including bacteraemia and meningitis (Black *et al*, 2000), and has secondary beneficial effects on pneumonia and otitis media through herd immunity (Grijalva *et al*, 2006; Shea *et al*, 2011). However, this protection is serotype specific, does not include acute bacterial sinusitis, and the effects on AOM and pneumonia are modest (Cutts *et al*, 2005; Shapiro *et al*, 2011). Thus, there is presently great interest in the development of serotype independent pneumococcal vaccines that can prevent the entire spectrum of pneumococcal-associated disease.

Live attenuated pneumococcal vaccines could represent a viable alternative to polysaccharide-based vaccines, since desirable, serotype independent responses to pneumococcal proteins might be generated in the context of a natural, mucosal infection. To achieve attenuation, Roche *et al* adopted a strategy of deleting the capsule, pneumolysin and pneumococcal surface protein A (Roche *et al*, 2007). While this strategy indeed conferred excellent protection against subsequent challenge, the deletion of antigenic virulence determinants may reduce potential efficacy as protective epitopes are lost. Indeed, protein-based vaccines in development focus on these very targets (McCool *et al*, 2003). Thus, we chose an attenuation strategy focused on microbial adaptation physiology with the reasoning that a short period of colonization with bacteria bearing the full complement of virulence determinants might be successful in mediating protection in the mucosa. The protection conferred by one of our live vaccine candidates was robust against multiple strains of pneumococcus, preventing AOM, sinusitis, invasive bacteraemia and pneumonia, even in the presence of influenza virus coinfection. Excellent protection was observed for both homologous and heterologous challenges indicating that this vaccination strategy could be used to confer broad, serotype independent protection against many forms of pneumococcal disease. The comparison of the *ftsY*-based vaccines to the *caxP*-based vaccine candidates suggests several features of an optimal mucosal vaccine. All four vaccines elicited high titer antibody but only the BHN97Δ*ftsY* vaccine was protective. This vaccine differed from the others in that it showed a more prolonged period of colonization, higher pneumococcal specific antibody titers, and a different isotype distribution dependent on CD4^+^ T-cell help.

Optimal protection against pneumococcal AOM in this study required intact CD4^+^ T cells at the time of vaccination and correlated with class switching to the mouse IgG2a, IgG2b and IgA serum antibody isotypes. Depletion of T cells at the time of challenge in vaccinated mice also had an effect on protection, but it was more modest than depletion at the time of vaccination such that the differences did not reach statistical significance (Fig 9). It should also be noted that there could have also been residual T-cell depletion during challenge in the groups being depleted at the time of vaccination, as depletion with the GK1.5 antibody can last for three to four weeks. Thus, while we can definitely show a role of CD4^+^ T cells in the class-switching phenotype, direct involvement in protection from AOM in this model remains unclear. The IgG2 isotypes in mice correspond to IgG1 in humans, as they are the FcR-interacting subclass (Daeron, 1997). We speculate that antibody-mediated opsonophagocytosis by mouse IgG2 isotype antibodies (IgG1 in humans) may be sufficient for clearance of pneumococci from the bloodstream and thus prevention of invasive disease, but FcR interactions may be required for elimination of bacteria from mucosal surfaces such as the lung, sinuses and middle ear. This may partially explain the effectiveness of the PCV against invasive pneumococcal disease but not mucosal infections (Eskola *et al*, 2001). It should be noted that we did not assess the impact of live, attenuated vaccination on levels of mucosal IgA, which could also be a contributing protective factor. Our findings regarding the importance of CD4^+^ T cells has a clinical correlation, as it has been shown that reduced functional CD4^+^ T-cell help is associated with susceptibility to recurrent otitis media in children (Sharma *et al*, 2011). A vaccine that induces better FcR interacting antibodies might help to overcome this defect, though as the BHN97Δ*ftsY*vaccine also requires CD4^+^ T cells, additional approaches will be will need to be considered for such patients.

Although the vaccine described in this report is very promising, several additional issues will need to be examined before it is a viable candidate for trials in humans. One major drawback to generating an effective and safe live, attenuated pneumococcal vaccine is that pneumococci are naturally competent. This feature allows for the risk of genetic recombination between the vaccine strain and the normal host flora, and potentially reversion to pathogenicity. To circumvent this possibility, the competence system should be deleted once an optimal attenuated vaccine strain has been generated. In addition, replacement of the pneumolysin gene with a non-toxic version incapable of damaging host tissues but retaining immunogenicity would also be of consideration. The allelic replacement with a toxoid version of pneumolysin is unlikely to affect the immunogenicity of the live vaccine, as such toxoid forms are both immunogenic and protective in against multiple serotypes during invasive pneumococcal infection (Alexander *et al*, 1994) capacity to damage the host.

## Materials and Methods

### Ethics statement

All experiments involving animals were performed with prior approval of and in accordance with guidelines of the St. Jude Institutional Animal Care and Use Committee. The St Jude laboratory animal facilities have been fully accredited by the American Association for Accreditation of Laboratory Animal Care. All chinchilla infection protocols were approved by the Wake Forest University Health Sciences Institutional Animal Care and Use Committee. Laboratory animals are maintained in accordance with the applicable portions of the Animal Welfare Act and the guidelines prescribed in the DHHS publication, Guide for the Care and Use of Laboratory Animals.

### Bacterial and viral strains and growth conditions

The TIGR4 (serotype 4), D39x (serotype 2) (Francis *et al*, 2001), BHN54 (serotype 7F, ST191) (McCullers *et al*, 2010), 6A4 (serotype 6A) (Lizcano *et al*, 2010) and BHN97 (serotype 19F, ST425, also known as SME33) (McCullers *et al*, 2007; McCullers *et al*, 2010) parent and mutant pneumococcal strains were grown overnight at 37°C in a 5% CO_2_ humidified incubator after being inoculated onto tryptic soy agar (TSA) plates supplemented with 3% sheep blood. Strains were then inoculated directly into semisynthetic liquid culture (CY broth) and grown to log phase before being administered to mice. The St. Jude strain of mouse adapted influenza virus A/Puerto Rico/8/34 (H1N1; PR8), generated by reverse genetics (McAuley *et al*, 2007), was grown in Madin-Darby canine kidney (MDCK) cells.

### Generation of mutants

Stable mutations in *caxP* (encoded by *SP1551*) and *ftsY* (encoded by *SP1244*) were generated by PCR SOEing as previously described (Horton, 1995). Primers used to amplify the flanking sequences are indicated in supplementary Table S1. Briefly, the coding region for*caxP* or *ftsY* was replaced with an erythromycin-resistance cassette by using homologous recombination. Transformants were selected on TSA plates supplemented with 3% sheep blood and erythromycin (1 mg/ml) after an overnight incubation at 37°C in a 5% CO_2_ humidified incubator. The *ftsY*- and *caxP*-strains in both backgrounds render the pneumococci avirulent, with at least a 3-log difference in LD_50_ compared to the parental strains. At the highest dosages of 10^8^ CFUs (colony-forming units) we observed survival greater than 90% for all live-attenuated strains. Insertion of the erythromycin cassette does not significantly impact pneumococcal virulence, as previously observed (Mann *et al*, 2012).

### Vaccination protocol

7 week old BALB/cJ mice anesthetized with 2.5% inhaled isoflurane were vaccinated with 10^5^ CFUs of the respective mutant strains in a volume of 25 μl phosphate-buffered saline (PBS) intranasally. Mock treated animals received PBS carrier alone. Killed bacteria (BHN97) at an equivalent CFU count were heat killed and then inoculated intranasally. PCV7 (Wyeth Pharmaceuticals Inc), PCV13 (Wyeth Pharmaceuticals Inc) and Pneumovax (PPV23, Merck and Co., Inc) vaccines were commercially acquired and were diluted in saline 1:10 and 100 μl was administered by intraperitoneal injection. After 4 weeks mice were boosted twice at 2 week intervals. Serum was collected 1 week following the final boost and mice were challenged two weeks after the final boost. Vaccination regimens were the same for all experimental conditions with all mice receiving three total vaccinations.

### Mouse challenge

To study AOM, groups of mice anesthetized with 2.5% inhaled isoflurane (*n* = 10–31; see figure legends for details of each experiment) were challenged intranasally with 10^5^ CFUs of BHN54 or BHN97 in 100 μl PBS as described previously (McCullers *et al*, 2007). To model invasive disease, mice were challenged intranasally with 1 × 10^7^ CFUs of TIGR4, 2.5 × 10^7^ CFUs D39 or 2 × 10^7^ CFUs of 6A4 in 25 μl PBS. The D39, BHN54 and BHN97 challenge strains had been engineered to express luciferase as described (Francis *et al*, 2001). In experiments involving influenza, PR8 was given intranasally in a volume of 100 μl of sterile PBS at a dose of 30 TCID_50_ 7 days prior to bacterial challenge (McCullers, 2004). For passive protection experiments, 200 μl serum from BHN97ΔftsY vaccinated animals was administered 1 h prior to challenge.

### Chinchilla challenges

Healthy adult chinchillas (*Chinchilla lanigera*) were purchased from Rauscher's Chinchillas (Larue, OH, USA) and allowed to acclimate to the vivarium for 1 week prior to infection or vaccination. All animals were examined by otoscopy prior to infection to ensure no visible signs of disease. Degree of otoscopic disease during infection was monitored and scored as follows: 0 = none, 1 = mild, 2 = moderate, 3 = frank purulence, 4 = tympanic membrane rupture. Chinchillas were anesthetized with isofluorane and inoculated via intranasal administration of 10^6^ CFUs. Animals were monitored daily and examined by otoscopy every 48 h to check for signs of disease as per the protocol approved by the Wake Forest Institutional Animal Care and Use Committee. For vaccination experiments, chinchillas were vaccinated via the intranasal route with 5 × 10^4^ CFU of BHN97Δ*ftsY* and boosted at 3 and 5 weeks. Two weeks following the final boost, animals were challenged with 9.5 × 10^4^ CFUs of the BHN97x strain by intranasal inoculation. At 7 days postchallenge middle ear bullae were aseptically removed, homogenized in PBS, serially diluted and bacterial load assessed by plate counting.

### Monitoring disease

The mice were monitored for AOM and sinusitis twice daily starting 6 h postchallenge and continuing until 72 h postchallenge. To monitor progression of disease, mice were anesthetized with 2.5% inhaled isoflurane before *in vivo* images of their left and right sides were taken as previously described (McCullers *et al*, 2007). During experiments modelling invasive disease, the bacterial burden in the bloodstream was measured by counting the CFUs formed by serial dilutions of blood collected from the mice. Mice were monitored daily for signs of infection. The mice were also monitored for weight loss over the entire challenge period for experiments involving influenza.

### Histology

Mice were euthanized at 24 or 72 h postinfection, and immediately perfused with 10% buffered formalin (Thermo Scientific, Kalamazoo, MI, USA) via the left cardiac ventricle. Additional formalin fixative was gently infused by syringe into the nasal passages and then the intact heads were post-fixed by immersion in 10% buffered formalin for an additional 48 h before being decalcified in formic acid (TBD-2 Decalcifier, Thermo Scientific, Kalamazoo, MI, USA). Multiple coronal sections of the head at the level of the ears and nasal passages were trimmed and embedded in paraffin, and 5 μm-thick sections were prepared and stained with hematoxylin and eosin for evaluation of inflammatory and degenerative lesions in the nasal passages, sinuses and middle ear.

### ELISAs

To measure serum antibody titers against different pneumococcal serotypes, bacterial strains were grown in C + Y broth until their optical densities at 620 nm were 0.5. Strains were diluted serially in 0.1 M carbonate buffer (pH 9.6) and transferred to 96-well ELISA plates (NUNC). The plates were spun at 2000*g* for 10 min before the supernatant was removed. The plates were dried under a vent hood for 1 h before unbound antibody surface were blocked in 10% FBS for 2 h. The plates were then washed three times with wash buffer (1% Tween-20, 1 mM Tris, 154 mM NaCl). Mouse serum from vaccinated animals was serially diluted in 10% FBS before it was added to the wells for 1 h, washed five times and incubated with AP-anti-mouse IgG (Southern Biotech) (1:2000) for 1 h. The plates were washed five times and then incubated 20 min in AP-yellow one component microwell substrate (Sigma) before measurements of their optical densities at 405 nm were taken in a Spectramax 340 plate reader (Molecular Devices). To measure serum antibody levels against specific proteins, recombinant proteins were all expressed in*Escherichia coli* and purified over a Ni++ column by the St Jude Protein Production Facility. The proteins utilized were recombinant choline binding protein A (rCbpA: amino acids 175-443 of SP2190 from TIGR4), recombinant pneumococcal surface protein A (rPspA: amino acids 1-302 from strain Rx1), and recombinant pneumolysin (PLY: amino acids 1-472 from D39). 100 ng of protein were utilized to coat the plates as described above.

### CD4 depletions

Mice were depleted of CD4^+^ T cells 48 h prior to vaccination, the day of vaccination, and 48 h postvaccination by IP injection of CD4-specific antibodies as previously described (Wanzeck *et al*, 2011). Depletions were undertaken both for the initial vaccination as well as for each subsequent boost. For CD4^+^ depletion at the time of challenge, mice were injected with ascites fluid containing the CD4-specific monoclonal antibody (MAb) GK1.5 commencing 3 days before infection and continuing at 2 day intervals thereafter as previously described (Riberdy *et al*, 1999). The efficacy of the protocol was checked at time of sampling by flow cytometric analysis (anti-CD4-PE) to confirm <1% of the respective cell population was present in blood. Depletion by this methodology results in >98% depletion in the periphery, lungs and spleen and has been utilized previously to discern the role of CD4^+^ T cells in pneumococcal interactions in the mucosa (Arora *et al*, 2006; Zhang *et al*, 2009).

### Immunoglobulin subtyping

Mouse serum from vaccinated and mock-treated animals was collected 96 h postchallenge as a terminal bleed. The serum antibodies were subtyped by using the Millipore Mouse Immunoglobulin Isotyping Kit according to the manufacturer's instructions.

### Statistical analyses

Comparison of survival between groups of mice was done with the Log Rank chi-squared test on the Kaplan–Meier survival data. Comparison of antibody titers and weight loss was done using analysis of variance (ANOVA). Comparison of proportions of otitis media, sinusitis, and pneumonia were done with the Chi-squared test with corrections for multiple comparisons. A *p*-value of <0.05 was considered significant for these comparisons. SigmaStat for Windows (SysStat Software, Inc., V 3.11) was utilized for all statistical analyses.

## Author contributions

JWR, ARI, JH, BM, GG, KM, AP, WS and MM performed the experiments. PV performed histological examination of tissues. JWR, EIT, JAM designed the experiments. JWR, ET and JAM wrote the manuscript.
