# Supplementary material for: A live-attenuated pneumococcal vaccine elicits CD4+ T-cell dependent class switching and provides serotype independent protection against acute otitis media
Source: EMBO Mol Med. 2013 Nov 4;6(1):141–54. doi: 10.1002/emmm.201202150 (PMC3936495; doi:10.1002/emmm.201202150)
Supplement: Supplementary file 1 [file emmm0006-0141-sd1.pdf]

EMBO Molecular Medicine

A Live-Attenuated Pneumococcal Vaccine Elicits CD4+ T-cell Dependent Class Switching and Provides Serotype Independent Protection Against Acute Otitis Media

Jason W. Rosch, Amy R. Iverson, Jessica Humann, Beth Mann, Geli Gao, Peter Vogel, Michael Mina, Kyle A. Murrah, Antonia C. Perez, W. Edward Swords, Elaine I. Tuomanen, and Jonathan A. McCullers

Corresponding author: Jason Rosch, St Jude Children's Research Hospital

Review timeline:

Submission date:	12 October 2012
Editorial Decision:	03 December 2012
Additional Author Correspondence:	05 December 2013
Revision received:	06 September 2013
Accepted:	23 September 2013

Transaction Report:

(Note: With the exception of the correction of typographical or spelling errors that could be a source of ambiguity, letters and reports are not edited. The original formatting of letters and referee reports may not be reflected in this compilation.)

Editors: Natasha Bushati / Céline Carret

1st Editorial Decision 03 December 2012

Thank you for the submission of your manuscript to EMBO Molecular Medicine. We have now heard back from the three referees whom we asked to evaluate your manuscript. As you will see from the reports below, referees 1 and 3 acknowledge the relevance and interest of your work.

Reviewer 2 is concerned about the novelty of the presented findings, but acknowledges that an alternative approach is used and protection against additional types of infection is described. We have consulted an additional expert advisor on this issue and consider these factors sufficiently novel for further consideration of your study.

All reviewers raise significant technical concerns. We would be open to consider a revised manuscript that addresses convincingly all the issues raised, with additional experimentation where appropriate.

Reviewer 2 suggests addressing whether the vaccine can protect against colonization, and to demonstrate the safety of the vaccine using IP challenge in mouse. We would like to ask you to address these points experimentally.

Reviewer 3 is concerned whether the mouse model used is sufficient to study otitis media and suggests using the chinchilla model. The expert advisor agrees that testing your approach in the chinchilla model would be important. We would therefore require you to test your vaccine in the suggested model.

In addition, both reviewer 3 and the expert advisor highlight that the number of strains tested is too low to claim heterologous protection. We therefore strongly encourage you to include more virulent strains as reviewer 3 suggests.

Finally, all three reviewers raise a number of important suggestions to improve the interpretation of your data, the impact and readability of your work and to enhance its translational potential.

Given the potential interest of your study, we would be willing to consider a revised manuscript with the understanding that the reviewers' concerns must be fully addressed, with additional experiments where appropriate.

Please note that it is EMBO Molecular Medicine policy to allow a single round of revision in order to avoid the delayed publication of research findings. Consequently, acceptance or rejection of the manuscript will depend on the completeness of your responses included in the next version of the manuscript.

EMBO Molecular Medicine has a "scooping protection" policy, whereby similar findings that are published by others during review or revision are not a criterion for rejection. I understand that the amount of work that would be required to submit a revised version of your manuscript is significant, hence, should you decide to submit a revised version, I do ask that you get in touch after three months if you have not completed it, to update us on the status.

Please also contact us as soon as possible if similar work is published elsewhere. If other work is published we may not be able to extend the revision period beyond three months.

I look forward to receiving your revised manuscript.

***** Reviewer's comments *****

Referee #1 (Comments on Novelty/Model System):

This is a well-designed, novel study, which has the potential to make a major contribution to pneumococcal vaccine development.

Referee #1 (Remarks):

This is a well-written manuscript describing a novel approach to development of a live attenuated, non-serotype-dependent pneumococcal vaccine. Whereas previous attempts have involved deletion of classical virulence factor genes, the authors of the current study point out that such deleted factors are likely to be important immunogens. Accordingly, they have targeted house-keeping genes that arerequired for long-term nasopharyngeal colonization. Choice of strain background was important, however, as only one of two genetic backgrounds tested, and only one of the targeted mutations (ftsY), resulted in a vaccine strain that could colonize the nasopharynx sufficiently, and for long enough to trigger protective, CD4+ T cell-dependent immune responses. This is a significant study and I have only minor comments for the authors attention:-

1. Line 123-4. It is not true to state that the fstY deleted strains were able to colonize for "several days", at least for the D39 background, as Fig 1 shows colonization at day 1, but not at the next time point (day 3). Thus, the data only support the conclusion that colonization lasts at least 1 day. Similarly on line 126, it is not appropriate to say that the D39 strains colonized for "3 days or less".

2. Lines 128, 129 and elsewhere: what is D39x? How does this differ fron D39? This does not seem to be explained anywhere.

3. Fig. 2A needs error bars; Fig. 2D-G need scale bars.

4. Protective efficacy seems to be dependent on capacity to colonize the nasopharynx for at least 7

days, since the only vaccine to show significant protection is the BHN97ftsY mutant. The authors should comment in the Discussion on the implications for, and lessons from, human disease, where periods of carriage do not necessarily result in protective homotypic or heterotypic immunity.

Referee #2 (Remarks):

General: This is a well-written, straightforward manuscript using well-characterized and established methods to demonstrate a novel live-attenuated pneumococcal vaccine that elicits serotype-independent, partial protection against mucosal pneumococcal disease, including otitis media. Protection correlates with the type and quantity of antibody elicited, and CD4+ T-cells are required for the humoral mediated protection. Moreover, the protection occurred in the presence of influenza virus, an important and common clinical co-infection.

Major

1) This is not the first description of a live-attenuated vaccine approach to pneumococcal infection demonstrating serotype-independent protection. The demonstration of protection against otitis media is novel but incremental. The premise that use of a regulatory gene knock out (ftsY) is better because it allows for immunity to virulence factors is conjecture. In the end, the authors state that it will be necessary to use a pneumolysin mutant. Since the protection is also independent of capsule type, neither of the two major virulence factors are important in the end anyway.
2) The success of the current pneumococcal vaccines has largely been attributed to the effect on colonization and herd immunity. Therefore it would be interesting to see if this vaccine can also protect against colonization, especially since the protection demonstrated in this study is focused on mucosal infections.
3) The use of the caxP mutant, which is soon dropped in the narrative, is a distraction and not useful.
4) Data on the requirement for CD4+T cells is completely expected if antibody is important and so this section is overstated.
5) The importance of isotype differences is conjecture and not proven to be important in the protection demonstrated. This is also overstated.
6) A more thorough demonstration of safety is needed. IP challenge would be a more rigorous test than provided in Fig 1.

Minor

1) Figure 1C x-axis reads "days post infection". Assume this should be "hours post infection"?
2) There is varying use of strains D39 and D39x but these are not the same -something not documented here. Example Fig uses D39x but Fig. 2 uses D39.
3) Is AOM diagnosis only determined by bioluminescence? Figure 2C, the diagnosis of sinusitis by bioluminescence - is this perhaps just colonization rather than sinus infection?
4) Color key in Figure 3 is not consistent (mock is black in some and pink in others)
5) Methods missing for passive protection experiments in Figure 3 E/F. How much serum was required for this protection?
6) Figure 5 is too confusing - need clearer and more concise way to graph data. Also, OD 405 not 450 in Figure 5 heading. In fact, Figure 5 and 6 could be combined to demonstrate overall role of antibody.
7) Pneumovax in x-axis of Supplemental figure S2 should be changed to PPV for consistency.
8) Mechanism of the traditional polysaccharide vaccines is not known - but is speculated to be through binding and opsonophagocytosis (intro correction)
9) Add "data not shown" to line 160, in regards to cross protection against AOM.
10) Children with reduced functional T-cell help have increased recurrent otitis infections - will this vaccine work in this population if CD4+ T-cells are reduced? Lines 278- 282
11) Methods state that influenza virus was administered in 100ul dose. Is this correct? Large volume for mouse nares, and would suggest virus is directed to lungs.
12) Line 371: antibody sites - change to "surface"
13) Depleting CD4+ T-cells does not completely remove vaccine induced protection, suggesting CD4+ T-cells switching of antibody subclass alone is not required for protection.
14) Confused by section 272 - 277. Why does this infer FcR required for mucosal protection? This

is too speculative.
15) PCVs show some efficacy against AOM. Change not to less in line 52.
16) Lines 267-270 are unclear.

Referee #3 (Comments on Novelty/Model System):

see comments below

Referee #3 (Remarks):

This manuscript report the design and testing of live attenuated pneumococcal vaccines in mouse models of infection and their ability to protect against acute otitis media, sinusitis and pneumonia.

There are some major points that should be taken into account before publishing this work.

1. Since protection from otitis media is the major goal of this work, I am questioning whether the mouse model is the best model to study this type of pneumococcal disease (what about chinchilla model? The choice should at least be discussed).
2. The reason why BHN97delta-FtsY is protective, while BHN97delta-caxP is not, is not clear from the data presented. In the discussion the authors claim that expression of the main antigens is not the same in these two strains but this is not clearly supported by Figure S1.
3. Why deletion of ftsY in the two strains (BHN97 and D39) results in two vaccines with completely different efficacy? This should be better discussed.
4. Heterologous strains used for challenge should be better characterized for their main characteristics (PspA and PspC variant expressed, expression of pili, etc)
5. Figure 3 panel C and D: maybe the dose used in this experiment should be adjusted as 40-50% protection is achieved with the mock vaccine as well.
6. It would be important to discuss more extensively about the possible implication of reversion to pathogenicity. Knocking out a single gene is probably not the best way to go when you work with a highly pathogenic organism. Pneumolysin should definitely be either knocked out or detoxified.
7. Results reported at page 7-8, lines 155-160 should be supported by a Figure. Maybe the experiment should be repeated using a more virulent strain and not a strain that only causes 50% infection.
8. In general, if authors want to strengthen the data on protection from heterologous challenge, more challenging strains should be used.

Minor points:

1. Figure 3E. Mock and PCV13 legends are inverted
2. Line 341. 100mL should read 100ul.

Additional Author Correspondence 05 December 2013

Thank you for both the comments of both the editorial board and the reviewers regarding our manuscript to ensure the strongest possible study in the final form.

We plan on addressing all the issues regarding the wording of the manuscript as well as all the murine challenges outlined in the reviews as these experiments can be readily addressed by our home institution, however we had one question that we would like to have addressed if possible.

Reviewer 3 suggested the chinchilla model to confirm these findings, or to at least address this issue in the discussion to which the expert advisor concurred that this would strengthen our study. While we agree with this, and could easily incorporate this into the discussion, we currently do not have either the expertise or animal approvals to accomplish these goals at our facilities (our animal use committee would require over 6 months to gain the required approvals for the use of chinchillas in

our BSL2 facility). To this end, we have been reaching out to other investigators that could potentially collaborate with such studies, with some success in indentifying interested parties, however the necessary pathogen approvals and experiments may take up to 6 months to accomplish given the number of variables that would need to be addressed (vaccine dosage, timing, challenge route, monitoring infection with our strain) though we are attempting to accomplish this as rapidly as possible.

Hence, our underlying query is to whether evidence of protection in the chinchilla model is a necessary experiment for the further consideration of the revised manuscript. If so, we will attempt to accomplish this by any means at our disposal, however it may require a significant extension for the time allocated for the revision, due the extensive nature of the experiments and institutional approvals required.

Thank you very much for your input into this matter,

1st Revision - authors' response 06 September 2013

Please find for your consideration the revised manuscript EMM-2012-02150 for which the editors graciously extended our revision deadline so that we could complete the requisite experiments to present the strongest possible manuscript. We have undertaken substantial additional experiments to strengthen the manuscript based on the comments of both the reviewers and the editorial board. This required a collaborative effort with the Swords laboratory, hence the inclusion of additional authors in this revision to reflect their important contributions to this study. In addition, the text has been modified to address clarity issues that were raised. Major additional work includes:

1. The use of the live vaccine to confer protective efficacy in a chinchilla model of otitis media. We were able to successfully utilize the BHN97 strain via an intranasal inoculation to induce otitis media in chinchillas. We demonstrate the live vaccine is protective in this additional animal model, further substantiating our claims of protection against otitis media.

2. Additional experiments into the safety of the ftsY based vaccine in an intraperitoneal model of infection.

3. Utilization of the live vaccine to confer protection against an additional invasive serotype of pneumococcus, further substantiating the observed serotype independent protection.

4. Demonstration of a greater antibody response to the live vaccine than to prolonged colonization by the parental strain.

5. Demonstration of protection against colonization with both homologous and heterologous challenges.

***** Reviewer's comments *****

Referee #1 (Comments on Novelty/Model System):

This is a well-designed, novel study, which has the potential to make a major contribution to pneumococcal vaccine development.

Referee #1 (Remarks):

This is a well-written manuscript describing a novel approach to development of a live attenuated, non-serotype-dependent pneumococcal vaccine. Whereas previous attempts have involved deletion of classical virulence factor genes, the authors of the current study point out that such deleted factors are likely to be important immunogens. Accordingly, they have targeted house-keeping genes that are required for long-term nasopharyngeal colonization. Choice of strain background was important, however, as only one of two genetic backgrounds tested, and only one of the targeted

mutations (ftsY), resulted in a vaccine strain that could colonize the nasopharynx sufficiently, and for long enough to trigger protective, CD4+ T cell-dependent immune responses. This is a significant study and I have only minor comments for the authors attention:

1. Line 123-4. It is not true to state that the fstY deleted strains were able to colonize for "several days", at least for the D39 background, as Fig 1 shows colonization at day 1, but not at the next time point (day 3). Thus, the data only support the conclusion that colonization lasts at least 1 day. Similarly on line 126, it is not appropriate to say that the D39 strains colonized for "3 days or less".

Response. We agree. We have reworked the text to more accurately represent the data as presented in Figure 1. We have also reworked Figure 1 to make the comparisons a more clear.

2. Lines 128, 129 and elsewhere: what is D39x? How does this differ from D39? This does not seem to be explained anywhere.

Response. D39x is a derivative of D39 with the luciferase cassette inserted. This is now included in the methods section and the original reference for the construction of D39x is now included in the methods section. We used D39x throughout these studies, and the figures and text have been updated accordingly.

3. Fig. 2A needs error bars; Fig. 2D-G need scale bars.
Response. Error bars and scale bars now included in the figure.

4. Protective efficacy seems to be dependent on capacity to colonize the nasopharynx for at least 7 days, since the only vaccine to show significant protection is the BHN97ftsY mutant. The authors should comment in the Discussion on the implications for, and lessons from, human disease, where periods of carriage do not necessarily result in protective homotypic or heterotypic immunity.

Response. This is an interesting point, which we get asked frequently, so we performed an additional experiment. Even though the BHN97x persists for long periods of time in nasal colonization (significant titters at 4 weeks) the serotype-independent antibody response to a single colonizing dose of this strain is much poorer than repeated inoculation of the vaccine strain. This can now be seen as supplementary Figure S5. We conclude that repeated dosing of a short duration colonizing strain confers greater antibody response than prolonged carriage. Lines 215-219.

Referee #2 (Remarks):

General: This is a well-written, straightforward manuscript using well-characterized and established methods to demonstrate a novel live-attenuated pneumococcal vaccine that elicits serotype-independent, partial protection against mucosal pneumococcal disease, including otitis media. Protection correlates with the type and quantity of antibody elicited, and CD4+ T-cells are required for the humoral mediated protection. Moreover, the protection occurred in the presence of influenza virus, an important and common clinical co-infection.

Major
1) This is not the first description of a live-attenuated vaccine approach to pneumococcal infection demonstrating serotype-independent protection. The demonstration of protection against otitis media is novel but incremental. The premise that use of a regulatory gene knock out (ftsY) is better because it allows for immunity to virulence factors is conjecture. In the end, the authors state that it will be necessary to use a pneumolysin mutant. Since the protection is also independent of capsule type, neither of the two major virulence factors are important in the end anyway.

Response. Our purpose for making the statement that the ftsY mutant would allow for immunity to various virulence factors is demonstrated in Supplementary Figure S3. Our main purpose in this statement was to indicate that by deleting a particular virulence gene that in of itself could be both immunogenic and protective could potentially reduce the efficacy of a live vaccine due to the loss of

one of the protective epitopes. We attempted to make this point more clearly in the manuscript to avoid confusion. Also, we suggest the use of a toxoid version of pneumolysin in the discussion rather than a complete deletion. Additional references which indicate that these non-toxic versions of pneumolysin as both immunogenic and protective against invasive disease are now included in the discussion.

2) The success of the current pneumococcal vaccines has largely been attributed to the effect on colonization and herd immunity. Therefore it would be interesting to see if this vaccine can also protect against colonization, especially since the protection demonstrated in this study is focused on mucosal infections.

Response. We undertook a colonization study with both a homologous and heterologous challenge using our live vaccine strain. We observed a significant decrease in nasal titters in the live vaccine group and this data is now included as Supplementary Figure S4.

3) The use of the caxP mutant, which is soon dropped in the narrative, is a distraction and not useful.

Response. The rationale for the inclusion of the caxP mutant was to demonstrate that dosing the mice with a mutant with no colonization ability resulted in no protection against otitis media, providing evidence that the only strain conferring protection colonized longer than the other 3 live attenuated vaccines. We also found it interesting that while all 4 live vaccines protected against weight loss, only one protected against disease. However, if this point remains a concern we are more than willing to delete these data from the manuscript.

4) Data on the requirement for CD4+T cells is completely expected if antibody is important and so this section is overstated.

Response. Language has been altered to de-emphasize this point and to limit our explanations to the interpretation of the data.

5) The importance of isotype differences is conjecture and not proven to be important in the protection demonstrated. This is also overstated.

Response. Language has been altered to clearly state that the protection correlated with isotype differences however we make clear that this may not be causative as there are likely other factors that play a role.

6) A more thorough demonstration of safety is needed. IP challenge would be a more rigorous test than provided in Fig 1.

Response. The wild type BHN97 strain does not readily cause invasive infection, hence we had to use a very high dose to observe mortality. Even so, the ftsY mutant vaccine remained highly attenuated in this IP model challenge which is now included in Figure 1.

Minor
1) Figure 1C x-axis reads "days post infection". Assume this should be "hours post infection"?

Response. Figure has been corrected.

2) There is varying use of strains D39 and D39x but these are not the same -something not documented here. Example Fig uses D39x but Fig. 2 uses D39.

Response. All experiments were performed with D39x. This has been corrected throughout the manuscript and the appropriate reference for the generation of the D39x strain along with all other strains utilized is now included in the methods section.

3) Is AOM diagnosis only determined by bioluminescence? Figure 2C, the diagnosis of sinusitis by bioluminescence - is this perhaps just colonization rather than sinus infection?

Response. In our initial studies with this model system we euthanized subsets of animals displaying AOM and sinusitis for histopathology. The histopathology results confirmed the observation of otitis media (Figure 2). To reduce the number of animals and to more readily collect data over multiple time points we relied mostly upon the xenogen imaging, supplemented by histopathology to confirm the findings.

4) Colour key in Figure 3 is not consistent (mock is black in some and pink in others)

Response. Colour scheme has been changed to be consistent throughout.

5) Methods missing for passive protection experiments in Figure 3 E/F. How much serum was required for this protection?

Response. Information is now provided in the methods section.

6) Figure 5 is too confusing - need clearer and more concise way to graph data. Also, OD 405 not 450 in Figure 5 heading. In fact, Figure 5 and 6 could be combined to demonstrate overall role of antibody.

Response. We opted for just showing one dilution of the antibody to reduce the amount of data shown in Figure 5.

7) Pneumovax in x-axis of Supplemental figure S2 should be changed to PPV for consistency.

Response. We have changed the figure accordingly.

8) Mechanism of the traditional polysaccharide vaccines is not known - but is speculated to be through binding and opsonophagocytosis (intro correction)

Response. Corrected as suggested.

9) Add "data not shown" to line 160, in regards to cross protection against AOM.

Response. Corrected as suggested.

10) Children with reduced functional T-cell help have increased recurrent otitis infections - will this vaccine work in this population if CD4+ T-cells are reduced? Lines 278- 282

Response. Likely not. This point has been made in the discussion adjacent to this statement.

11) Methods state that influenza virus was administered in 100ul dose. Is this correct? Large volume for mouse nares, and would suggest virus is directed to lungs.

Response. Yes this is correct as this has been the previously published method and we wished to be as consistent as possible with previously methodologies. As we were investigating pneumonia for this model, it was important that the virus reach both the mouse nares as well as the lungs.

12) Line 371: antibody sites - change to "surface"

Response. Corrected as indicated.

13) Depleting CD4+ T-cells does not completely remove vaccine induced protection, suggesting CD4+ T-cells switching of antibody subclass alone is not required for protection.

Response. We agree, and we allow for the possibility of additional factors in the protection in the discussion as well as the results.

14) Confused by section 272 - 277. Why does this infer FcR required for mucosal protection? This is too speculative.

Response. We merely wished to comment about how the immunoglobulin subtypes were distinct from the PCV vaccine and relate it to the clinical observation that while the PCV vaccine is protective against invasive disease, protection at the mucosal surfaces is not observed. We altered the text to clearly indicate this was speculation however, and provide an additional sentence immediately thereafter further explaining this rationale.

15) PCVs show some efficacy against AOM. Change not to less in line 52.

Response. We agree. Changed as suggested.

16) Lines 267-270 are unclear.

Response. We agree. Sentence has been modified for clarity.

Referee #3 (Comments on Novelty/Model System):

see comments below

Referee #3 (Remarks):

This manuscript report the design and testing of live attenuated pneumococcal vaccines in mouse models of infection and their ability to protect against acute otitis media, sinusitis and pneumonia.

There are some major points that should be taken into account before publishing this work.

1. Since protection from otitis media is the major goal of this work, I am questioning whether the mouse model is the best model to study this type of pneumococcal disease (what about chinchilla model? The choice should at least be discussed).

Response. This was a request that required significant time and effort, hence the delay in our resubmission. We were able to engage a collaborator with expertise in the chinchilla model and were able to complete this reviewer request. We initiated a study to determine if the BHN97 strain causes otitis media in chinchillas and were successful. Then we demonstrated that the BHN97 ΔftsY vaccine conferred effective protection in the chinchilla model of infection.

2. The reason why BHN97delta-FtsY is protective, while BHN97delta-caxP is not, is not clear from the data presented. In the discussion the authors claim that expression of the main antigens is not the same in these two strains but this is not clearly supported by Figure S1.

Response. The antigens are the same but the antibody responses differ because of the length of colonization. We suspect part of the reason is that the ftsY strain tends to colonize for longer periods of time compared to the caxP strain, resulted in significantly greater serotype independent antibody titters. We clarified the text to highlight this and to eliminate and potential confusion.

3. Why deletion of ftsY in the two strains (BHN97 and D39) results in two vaccines with completely different efficacy? This should be better discussed.

Response. We believe this to be the case because the BHN97 deletion tends to colonize for longer periods of time compared to the D39x variant. It should also be noted that the BHN97 may also encode different variants of virulence genes, as we observed with the non-reactivity of the strain using the monoclonal antibody against PspA that readily reacted with the D39x strain. There are also likely to be other important differences between these strains, as the D39x strain causes a robust pneumonia and sepsis in murine models whereas the BHN97 does not typically cause invasive disease.

4. Heterologous strains used for challenge should be better characterized for their main characteristics (PspA and PspC variant expressed, expression of pili, etc.)

Response. We have now included the original reference for each of the challenge strains utilized in the methods section.

5. Figure 3 panel C and D: maybe the dose used in this experiment should be adjusted as 40-50% protection is achieved with the mock vaccine as well.

Response. We attempted to achieve an LD50. We have performed additional experiments to increase the lethality of the mock challenge so that survival is closer to 20% by increasing the inoculum dosage. This is now reflected in the updated figure.

6. It would be important to discuss more extensively about the possible implication of reversion to pathogenicity. Knocking out a single gene is probably not the best way to go when you work with a highly pathogenic organism. Pneumolysin should definitely be either knocked out or detoxified.

Response. Detoxification will likely be needed as will deletion of the competence machinery to minimize the possibility of reversion. An additional sentence in the discussion in regards to the use of a toxoid pneumolysin still being able to confer protection is now included.

7. Results reported at page 7-8, lines 155-160 should be supported by a Figure. Maybe the experiment should be repeated using a more virulent strain and not a strain that only causes 50% infection.

Response. Data is included in Figure 4. Unfortunately many of our strains do not cause robust or consistent otitis media through the nasal passages in our laboratory. While we could not do this for the otitis experiment, we were able to show additional protection against an additional serotype during both invasive disease (Figure 4, panels E,F) and during colonization (Supplementary Figure S4) to strengthen our demonstration of serotype independent protection.

8. In general, if authors want to strengthen the data on protection from heterologous challenge, more challenging strains should be used.

Response. We have now included a serotype 6 strain in our study which we observed significant protection during invasive disease in terms of survival and bacterial burden in the blood following infection (see comment above).

Minor points:
1. Figure 3E. Mock and PCV13 legends are inverted

Response. Changed as suggested.

2. Line 341. 100mL should read 100ul.

Response. Changed as suggested.

2nd Editorial Decision	23 September 2013

Please find enclosed the final reports on your manuscript. We are pleased to inform you that your manuscript is accepted for publication and is being sent to our publisher to be included in the next available issue of EMBO Molecular Medicine.

***** Reviewer's comments *****

Referee #3 (Comments on Novelty/Model System):

I have read the revised version of the manuscript along with the point by point response and I acknowledge that most of the criticisms I initially raised have been addressed in the present form of the manuscript. In particular, the data obtained with the chinchilla model have now been included. Therefore, I believe that this paper is now ecceptable for publication in your journal.

Referee #4 (Remarks):

From my point of view this work is now technically well done and demonstrates in all possible small animal models the potential of this vaccine. In addition the vaccine proposed, being live-attenuated is an innovative concept in the field of pneumococcus. Obviously we do not know how the vaccine would perform in humans but this paper provides the necessary data to support testing it in man. I recommend publishing the paper.
