# Supplementary material for: A live-attenuated pneumococcal vaccine elicits CD4+ T-cell dependent class switching and provides serotype independent protection against acute otitis media
Source: EMBO Mol Med. 2013 Nov 4;6(1):141–54. doi: 10.1002/emmm.201202150 (PMC3936495; doi:10.1002/emmm.201202150)

Figure S1. Levels of expression of PspA, CbpA, and pneumolysin in the parental and vaccine strains. Data represents 1:3 serial dilution of monoclonal antisera beginning with a 1:50 dilution on the leftmost bars. Indpependent cultures of the respective strains were grown and used for ELISA with monoclonal antibodies against the indicated proteins. Note that no signal was observed with PspA in all the BHN97 strains (F), indicating the epitope for the monoclonal antibody is likely not conserved in this strain.

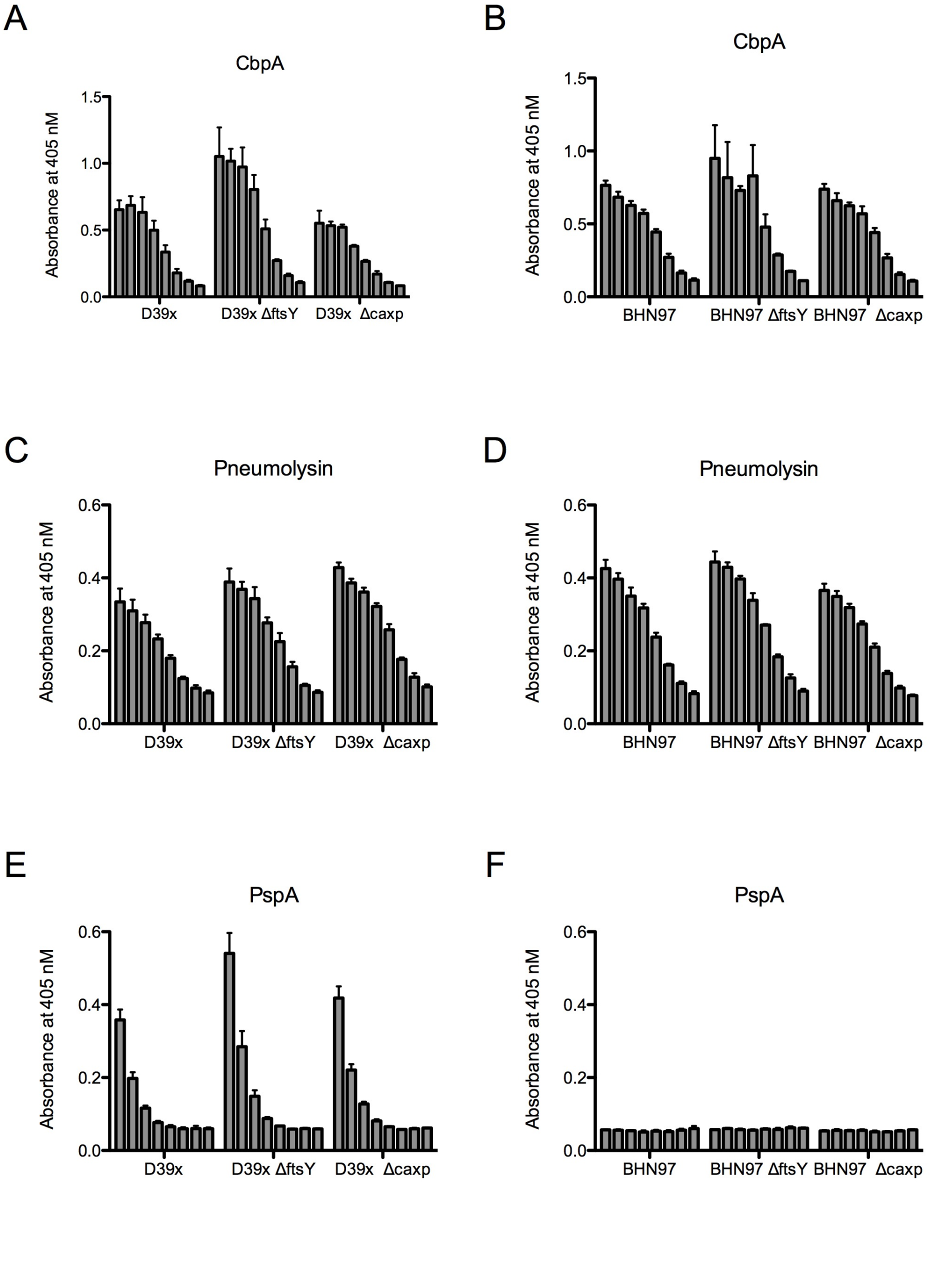

Supplement: Supplementary file 2 [file emmm0006-0141-sd2.pdf]
