# Supplementary material for: A live-attenuated pneumococcal vaccine elicits CD4+ T-cell dependent class switching and provides serotype independent protection against acute otitis media
Source: EMBO Mol Med. 2013 Nov 4;6(1):141–54. doi: 10.1002/emmm.201202150 (PMC3936495; doi:10.1002/emmm.201202150)

Figure S2. Luminscent intensity in the ears and sinuses at 24 and 72 hours post infection for the various vaccine conditions. Quantitation is as described in Figure 4.

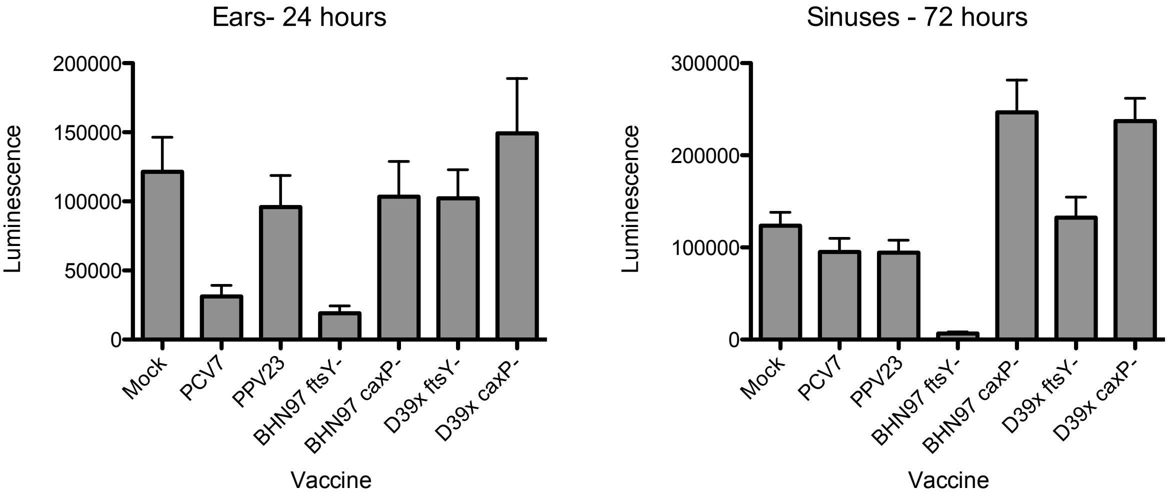

Supplement: Supplementary file 3 [file emmm0006-0141-sd3.pdf]
