# Supplementary material for: A live-attenuated pneumococcal vaccine elicits CD4+ T-cell dependent class switching and provides serotype independent protection against acute otitis media
Source: EMBO Mol Med. 2013 Nov 4;6(1):141–54. doi: 10.1002/emmm.201202150 (PMC3936495; doi:10.1002/emmm.201202150)

Figure S3. The BHN97 ftsY vaccination induces signficantly greater antibody titers than or intranasal heat killed BHN97. Serum antibody titers against Pneumolysin, CbpA, and PspA were determined by ELISA using purified recombinant protein.

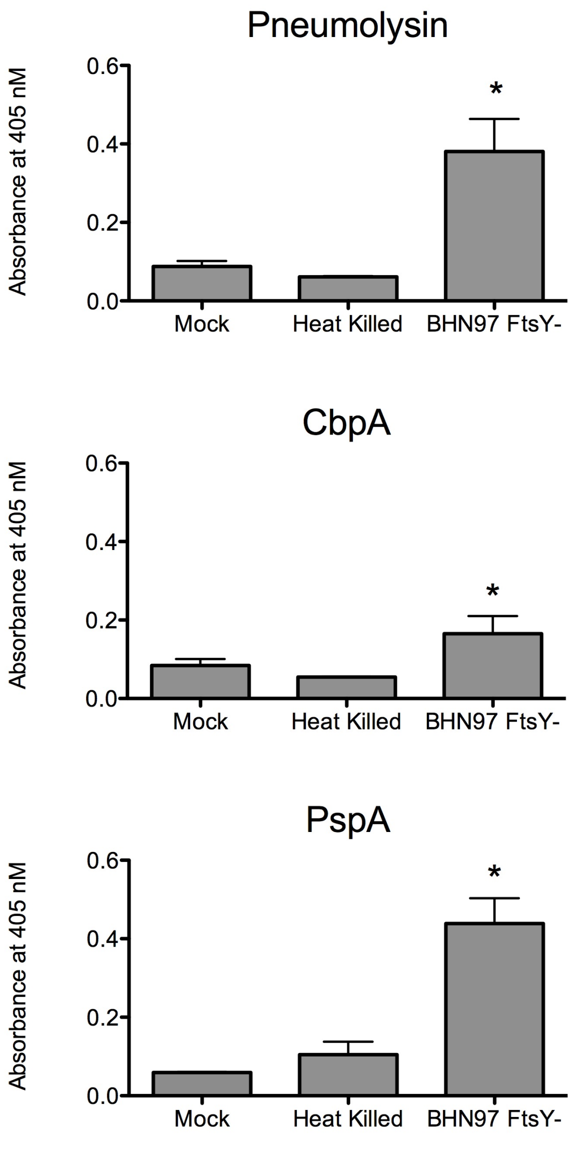

Supplement: Supplementary file 4 [file emmm0006-0141-sd4.pdf]
