# Supplementary material for: A live-attenuated pneumococcal vaccine elicits CD4+ T-cell dependent class switching and provides serotype independent protection against acute otitis media
Source: EMBO Mol Med. 2013 Nov 4;6(1):141–54. doi: 10.1002/emmm.201202150 (PMC3936495; doi:10.1002/emmm.201202150)

Figure S4. Live vaccine confers homologous and heterologous protection against colonization. Bacterial loads in the nasal passages of BHN97 mice (day 7 post challenge) and the 6A4 strain (day 3 post challenge) as determined by nasal lavage and plating for colony forming units. Due to the lethality of the 6A challenge, the day 3 time point is shown due to the low survival of the mock animals at day 7. *= $p<0.05$ by Mann-Whitney.

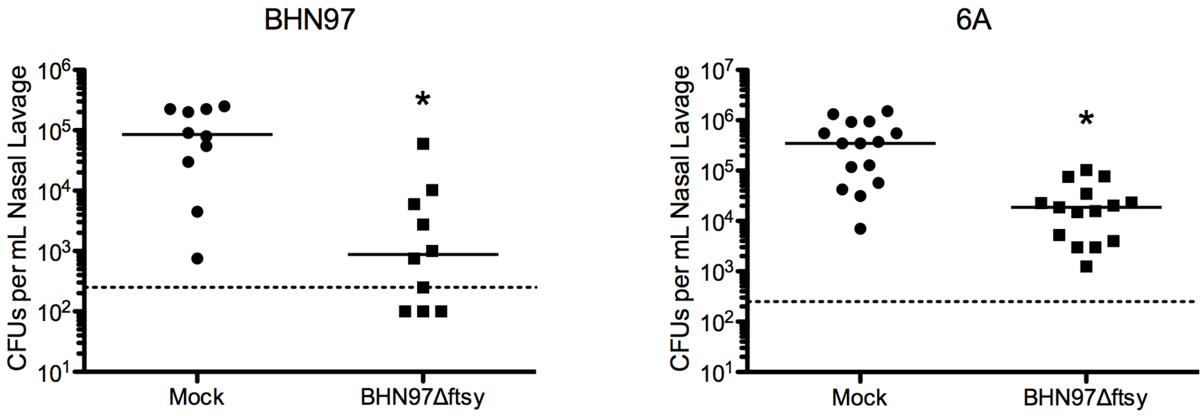

Supplement: Supplementary file 5 [file emmm0006-0141-sd5.pdf]
