# Supplementary material for: A live-attenuated pneumococcal vaccine elicits CD4+ T-cell dependent class switching and provides serotype independent protection against acute otitis media
Source: EMBO Mol Med. 2013 Nov 4;6(1):141–54. doi: 10.1002/emmm.201202150 (PMC3936495; doi:10.1002/emmm.201202150)
Supplement: Supplementary file 6 [file emmm0006-0141-sd6.pdf]

Figure S5. Live vaccine confers greater heterologous antibody titers than prolonger colonization with parental strain. Mice were either infected with a single dose doe the BHN97 strain, resulting in colonization for over 4 weeks, or subjected to the BHN97 ΔftsY vaccine regimen as described in the methods section. While both groups developed equivalent antibody titers against the parental 19F strain, the live vaccine resulted in significantly greater antibody titers against heterologous strains. *= p<0.05 by Mann-Whitney.

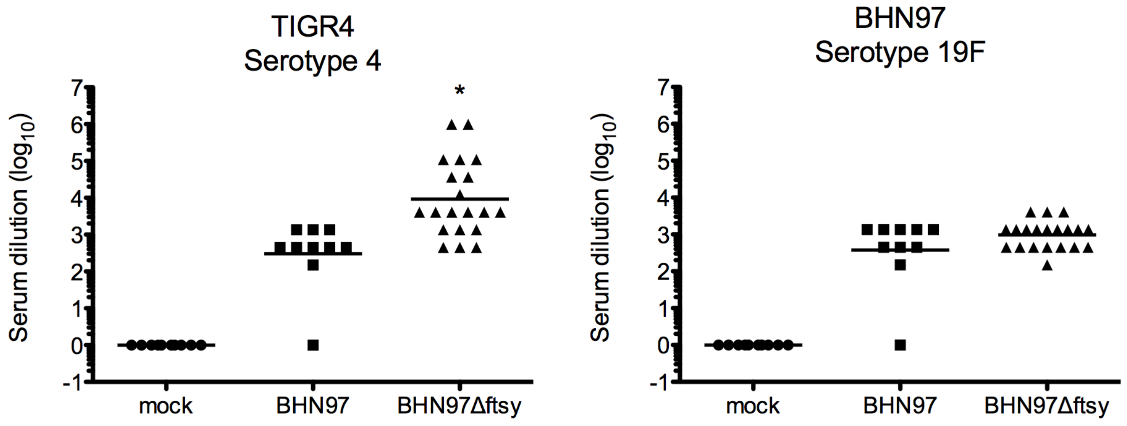

Table S1. Primers used in this study.

**Oligonucleotide**	**Sequence**
FTSY UP F	CGACCATTATCTTGGTAAGGAAATGATCC
FTSY UP R	GAGTCGCTTTTGTAAATTTGGTCAGAAGCAAAAATCCTGCAAGGCATAAAC
FTSY DOWN F	GTTTGCTTCTAAGTCTTATTTCCTTAGTTCTCCTTTAGCACATATTCTTC
FTSY DOWN R	GGCTTTGAATCCATTGATATTGTTCTGG
CAXP UP F	CGCATTCTTGTCAAAACCGAGTTTGCG
CAXP UP R	GAGTCGCTTTTGTAAATTTGGGCCATGACAGGTGACGGTGTCAATGACGCGCC
CAXP DOWN F	GTTTGCTTCTAAGTCTTATTTCCCCGCATCTTGAAGCATACCAGCAATATGACC
CAXP DOWN R	GGAGGAGACACATGTCAAAAGAACAAAAACGCCAAGCG
